# Small-molecule inhibition of the METTL3/METTL14 complex suppresses neuroblastoma tumor growth and promotes differentiation

**DOI:** 10.1016/j.celrep.2024.114165

**Published:** 2024-04-30

**Authors:** Monica Pomaville, Mohansrinivas Chennakesavalu, Pingluan Wang, Zhiwei Jiang, Hui-Lung Sun, Peizhe Ren, Ryan Borchert, Varsha Gupta, Chang Ye, Ruiqi Ge, Zhongyu Zhu, Mallory Brodnik, Yuhao Zhong, Kelley Moore, Helen Salwen, Rani E. George, Malgorzata Krajewska, Alexandre Chlenski, Mark A. Applebaum, Chuan He, Susan L. Cohn

**Affiliations:** 1 Department of Pediatrics, University of Chicago Comer Children’s Hospital, Chicago, IL 60637, USA; 2 Department of Chemistry, University of Chicago, Chicago, IL 60637, USA; 3 Department of Pediatric Hematology/Oncology, Dana-Farber Cancer Institute, Boston, MA 02215, USA; 4 School of Biochemistry and Cell Biology, Biosciences Institute, University College Cork, Cork, Ireland; 5 Howard Hughes Medical Institute, University of Chicago, Chicago, Il 60637 USA; 6 These authors contributed equally; 7 Present address: Department of Pediatric Oncology, Children’s Hospital of Philadelphia, Philadelphia, PA 19104, USA; 8 Lead contact

## Abstract

The *N*^6^-methyladenosine (m^6^A) RNA modification is an important regulator of gene expression. m^6^A is deposited by a methyltransferase complex that includes methyltransferase-like 3 (METTL3) and methyltransferase-like 14 (METTL14). High levels of METTL3/METTL14 drive the growth of many types of adult cancer, and METTL3/METTL14 inhibitors are emerging as new anticancer agents. However, little is known about the m^6^A epitranscriptome or the role of the METTL3/METTL14 complex in neuroblastoma, a common pediatric cancer. Here, we show that METTL3 knockdown or pharmacologic inhibition with the small molecule STM2457 leads to reduced neuroblastoma cell proliferation and increased differentiation. These changes in neuroblastoma phenotype are associated with decreased m^6^A deposition on transcripts involved in nervous system development and neuronal differentiation, with increased stability of target mRNAs. In preclinical studies, STM2457 treatment suppresses the growth of neuroblastoma tumors *in vivo*. Together, these results support the potential of METTL3/METTL14 complex inhibition as a therapeutic strategy against neuroblastoma.

## In brief

Pomaville et al. show that the catalytic activity of the METTL3/14 complex is critical for neuroblastoma growth. Pharmacological inhibition of METTL3/14 promoted neuroblastoma differentiation and suppressed tumor xenograft growth. Inhibitor treatment mediated m^6^A loss at sites specific for neuronal differentiation, increasing transcript stability and the expression of neuronal differentiation networks.

## Graphical abstract

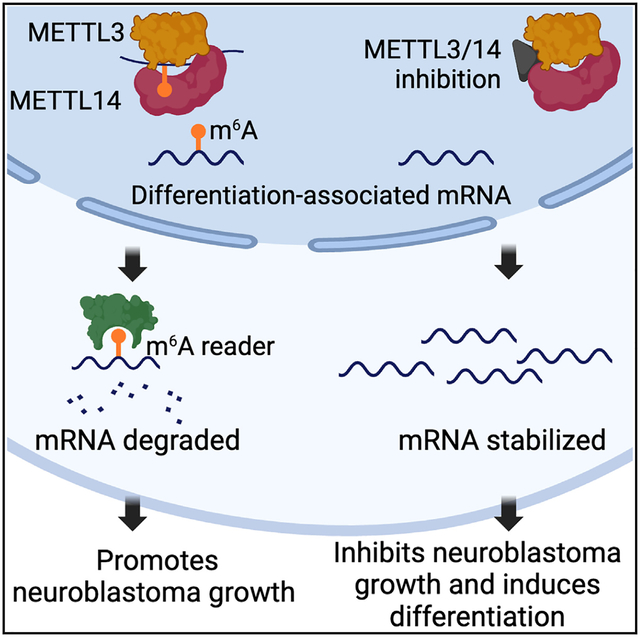

## INTRODUCTION

RNA modifications play critical roles in regulating gene expression. The most prevalent mRNA modification, *N*^6^-methyladenosine (m^6^A), regulates the fates of modified mRNA molecules and affects many important biological processes including development and stem cell differentiation. Aberrant m^6^A deposition is implicated in various diseases including cancer.^1^ This RNA modification offers cells transcriptome flexibility. Placement, removal, and reading of m^6^A occur throughout normal physiology to tune global gene expression and are promoted by stimuli such as cell stress or differentiation.^2,3^ m^6^A modifications are installed by writer proteins, removed by eraser proteins, and recognized by different reader proteins. Inhibitors of m^6^A modification proteins are emerging as novel anticancer agents.^4–7^

Although the methyltransferase-like 3 (METTL3) writer acts as a tumor suppressor in some cancer types, this methyltransferase has largely been found to have tumor-promoting functions.^4,8,9^ In many adult solid tumors, elevated METTL3 has been shown to function as an oncogene, and this enzyme has also been linked to the initiation and maintenance of acute myeloid leukemia (AML).^10^ In support of the therapeutic potential of targeting METTL3, genetic knockdown decreases cell proliferation and suppresses tumor growth in a number of adult cancer models.^10^ Further, STM2457, a selective first-in-class METTL3 inhibitor that targets the catalytic activity of the METTL3/METTL14 (METTL3/14) complex, has been shown to impair engraftment and prolong survival in mouse models of AML.^5^ Other small-molecule METTL3 inhibitors with considerable activity against a broad range of adult cancers have also been identified, and a first-in-human trial testing the METTL3 inhibitor STC-15 in adult patients with cancer is ongoing (ClinicalTrials.gov: NCT05584111).^10^ However, little is known about m^6^A RNA modifications or the role of METTL3 in pediatric cancers.

Neuroblastoma is a common pediatric cancer that originates in neural crest cells.^11,12^ Despite treatment with intensive, multi-modality therapy, long-term survival remains poor for children with clinically aggressive, high-risk disease.^11,13^ Thus, there is an urgent need for more effective strategies. Similar to other pediatric cancers, there is a paucity of recurrent genomic mutations in neuroblastoma, and epigenetic dysregulation plays a key role in determining neuroblastoma phenotype and cell lineage.^14–16^ Neuroblastoma cells classically exhibit plasticity between adrenergic and mesenchymal lineages, and recent reports show that both populations are present in neuroblastoma tumors.^14,17^ In this study, we investigated the effects of METTL3-dependent m^6^A RNA modifications on neuroblastoma gene expression and tumor growth. We also tested the antineuroblastoma activity of the METTL3 inhibitor STM2457. We show that small interfering RNA (siRNA)-mediated *METTL3* knockdown or STM2457 treatment decreased the viability of adrenergic neuroblastoma cells and promoted differentiation. STM2457 treatment also suppressed the growth of neuroblastoma tumors *in vivo*. Our results suggest that targeting METTL3 and other RNA modifying enzymes may have therapeutic potential in neuroblastoma.

## RESULTS

### The METTL3/14 complex is a potential therapeutic target in neuroblastoma

**Figure 1.**
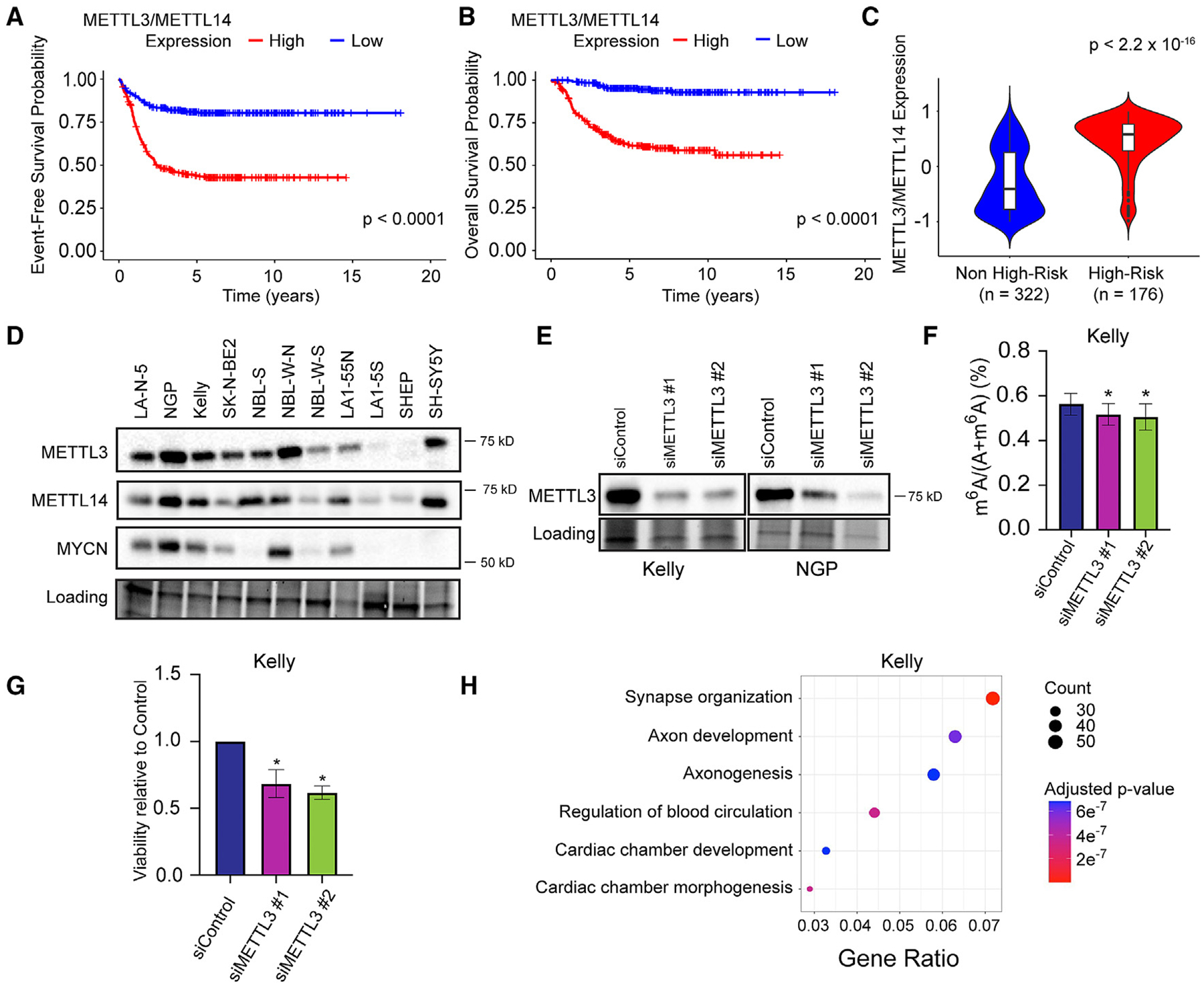
METTL3/METTL14 expression correlates with survival in patients with neuroblastoma, and METTL3 siRNA knockdown induces neuroblastoma cell differentiation (A and B) Event-free survival (A) and overall survival (B) are significantly inferior for patients in the SEQC-NB cohort (*n* = 498) with neuroblastomas that express high vs. low levels of METTL3/METTL14 expression by log-rank test (*p* < 0.05). (C) Violin plot showing high-risk patients in the SEQC-NB cohort had significantly higher METTL3/METTL14 expression compared to non-high-risk patients (*p* < 2.2 × 10^−16^, unpaired t test). (D) Western blot analysis shows heterogeneous levels of METTL3, METTL14, and MYCN expression among 11 neuroblastoma cell lines with gel activation image as loading reference. (E) METTL3 siRNA decreased METTL3 protein expression in Kelly and NGP neuroblastoma cells compared to control. (F) Global levels of m^6^A, quantified by triple-quadrupole liquid chromatography mass spectrometry (LC-MS QQQ), are decreased in neuroblastoma cells with siRNA-mediated METTL3 knockdown compared to control cells (*n* = 3, *p* < 0.05, unpaired t test). (G) Cell viability is decreased in neuroblastoma cells with siRNA-mediated METTL3 knockdown compared to controls (*n* = 3, *p* < 0.05, unpaired t test). (H) Up-regulated genes in Kelly cells with siRNA-mediated METTL3 knockdown were enriched for Gene Ontology (GO) pathways of synapse organization and axon development (*n* = 3 biological replicates). Bar graphs show mean and SD of data from 3 independent experiments.

m^6^A is installed onto mRNA by the METTL3/METTL14 methyltransferase complex.^18^ We evaluated the level of tumor expression of METTL3 and METTL14 in two independent neuroblastoma datasets. In both the SEQC-NB (*n* = 498, GEO: GSE49710) and Westermann (*n* = 105, GEO: GSE73518) cohorts, high expression of METTL3 and METTL14 was associated with significantly inferior event-free survival (EFS) (*p* < 0.0001 and *p* = 0.0041) and overall survival (OS) (*p* < 0.0001 and *p* = 0.0014), respectively (Figures 1A, 1B, S1A, and S1B). High-risk tumors also had significantly higher expression of METTL3 and METTL14 compared to non-high-risk tumors in the two cohorts (*n* = 498 patients, *p* < 2.2 × 10^−16^; *n* = 105 patients, *p* = 1.1 × 10^−6^) (Figures 1C and S1C). METTL3 and METTL14 protein levels were analyzed across the 11 human neuroblastoma cell lines (Figure 1D). Among the eight *MYCN*-amplified cell lines analyzed in this study, six have an adrenergic cell lineage phenotype (LAN-5, NGP, Kelly, SK-N-BE2, NBL-W-N, and LA1–55N) and two (NBL-W-S and LA1–5S) have a mesenchymal phenotype. Of the three non-*MYCN*-amplified cells lines evaluated, two have an adrenergic phenotype (NBL-S, SH-SY5Y), and one (SHEP) is mesenchymal. Adrenergic cell lines expressed higher levels of METTL3 and METTL14 proteins compared to mesenchymal cell lines (Figure 1D). To functionally characterize the effects of METTL3 in neuroblastoma, two siRNAs (siMETTL3#1 and siMETTL3#2) targeting *METTL3* were used in transfection experiments and compared to control siRNA. Western blot analysis demonstrated that both siRNA molecules mediated *METTL3* knockdown in Kelly and NGP cells (Figure 1E). Global m^6^A levels fell by approximately 8%–10% after 72 h of incubation with two siRNAs, as assessed by high-performance liquid chromatography coupled with triple quadrupole mass spectrometry (*p* = 0.0388, *p* = 0.0233, respectively) (Figure 1F). *METTL3* knockdown mediated by the two siRNAs decreased the viability of Kelly cells as measured by MTS assay compared to controls (*p* = 0.0092 and *p* = 0.0006, respectively) (Figure 1G). Further, *METTL3* knockdown affected gene expression in neuroblastoma cells, with up-regulated genes showing enrichment for pathways involved in neuronal differentiation such as axon development and axonogenesis, detected by RNA sequencing Gene Ontology Biological Pathway (GOBP) analysis (Figure 1H).

### METTL3/14 inhibition decreases adrenergic neuroblastoma cell proliferation and suppresses neuroblastoma tumor growth

**Figure 2.**
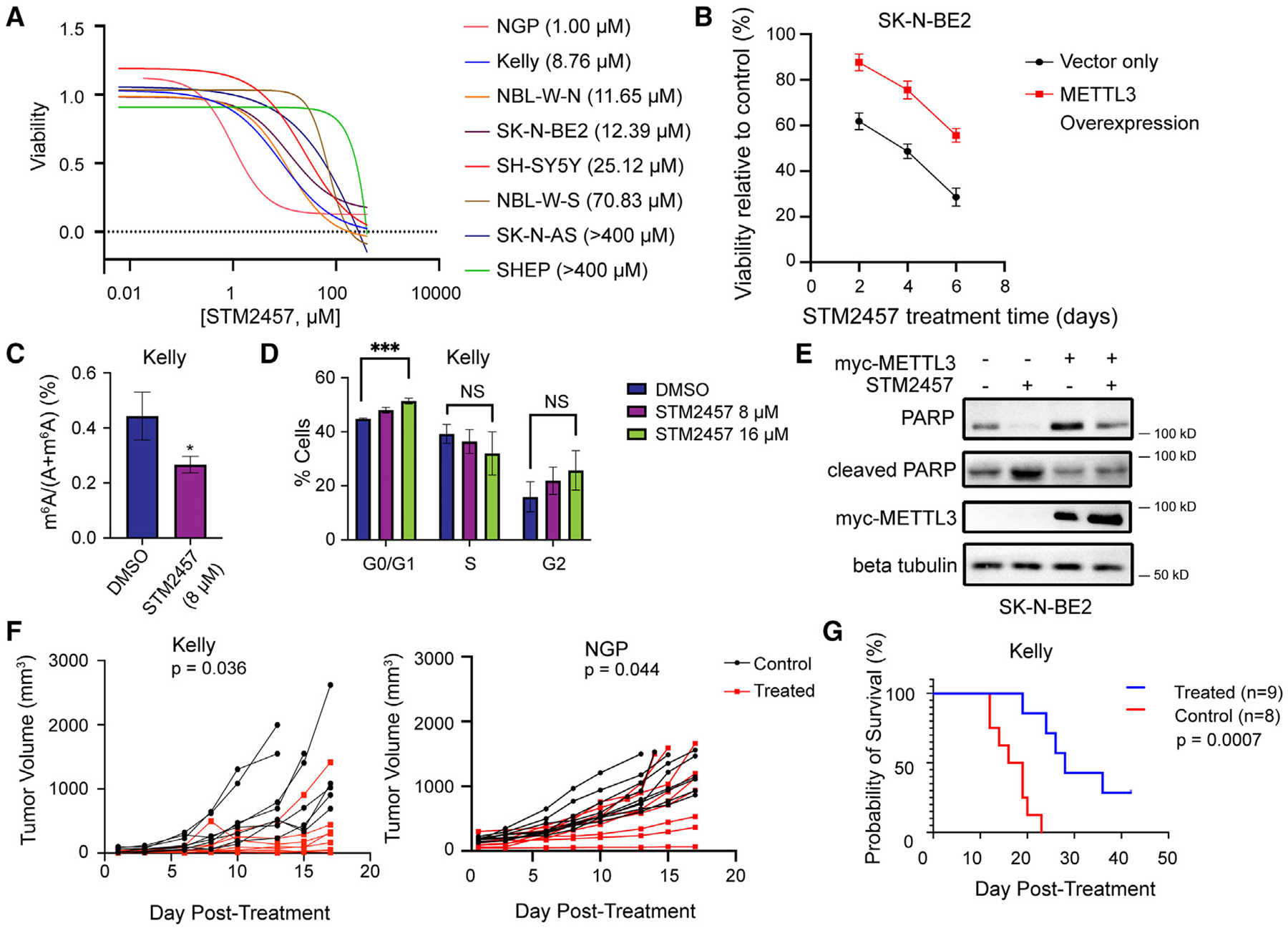
STM2457 treatment decreases neuroblastoma cell proliferation and global levels of m^6^A *in vitro* and impairs the growth of neuroblastoma xenografts *in vivo* (A) STM2457 dose-response curves for 8 neuroblastoma cell lines (mean curve of 3 biological replicates). (B) The viability of SK-N-BE2 cells with overexpression of METTL3 treated with 16 μM STM2457 is higher relative to control cells (*n* = 3, *p* < 0.05, unpaired t test). (C) Quantitative m^6^A analysis by LC-MS QQQ shows that global m^6^A levels are significantly decreased in Kelly cells treated with 8 μM STM2457 compared to cells treated with DMSO control (*n* = 3, *p* < 0.05, unpaired t test). (D) Cell cycle analysis shows that treatment with 16 μM STM2457 increases the distribution of Kelly cells in G0/G1 compared to DMSO (*n* = 3, *p* < 0.001, unpaired t test). (E) Western blot analysis shows that cleaved PARP expression is decreased in SK-N-BE2 cells with METTL3 overexpression treated with 16 μM STM2457 compared to control cells treated with STM2457. (F) STM2457 treatment (50 mg/kg/day × 14 days) suppresses the growth of subcutaneous neuroblastoma xenografts (mm^3^) comprised of Kelly cells (*p* = 0.036, unpaired t test) or NGP cells (*p* = 0.044) compared to vehicle control. (G) STM2457 treatment improves overall survival for mice compared to vehicle control in neuroblastoma xenografts comprised of Kelly cells (*p* = 0.0007, log-rank test). Bar graphs show mean and SD of data from 3 independent experiments.

METTL3 is the catalytic component of the METTL3/14 heterodimer, while METTL14 facilitates RNA target binding.^19^ METTL14 is required for METTL3 to exert effective catalytic function.^19^ Both METTL3 and METTL14 have functions independent of the methylation activity.^20,21^ To emulate the mechanism of METTL3/14 inhibition that would be leveraged therapeutically, the small-molecule METTL3 inhibitor STM2457 was used to target catalytic activity of the methyltransferase complex (Figure S7).^5^ The effect of STM2457 on cell growth was evaluated in eight neuroblastoma cell lines. The IC_50_ values ranged from 1.0 to 25.1 μM in the adrenergic cell lines (Kelly, NGP, NBL-W-N, NGP, SK-N-BE2, SH-SY5Y, LA1–55N) (Figure 2A). Mesenchymal cell lines (NBL-W-S, SK-N-AS, SHEP) were more resistant. Overexpression of METTL3 rescued the growth-inhibitory effects of STM2457 in SK-N-BE2 cells (Figure 2B). Treatment with STM2457 below the IC_50_ value of 8 μM led to a 40% decrease in m^6^A levels in Kelly cells within 24 h (*p* = 0.0294) (Figure 2C). Six days of STM2457 treatment at concentrations of 8 or 16 μM modified the cell cycle in Kelly cells, with an increase in G0/G1 by 3.3% (*p* = 0.0253) and 6.6% (*p* = 0.0005), respectively (Figure 2D). STM2457 treatment also increased the level of cleaved PARP protein in SK-N-BE2 cells, indicating that METTL3 inhibition induces apoptosis in neuroblastoma cells (Figure 2E). Overexpression of METTL3 reduced the level of cleaved PARP expression in cells treated with STM2457, supporting an on-target effect of the drug (Figure 2E). STM2457 also significantly suppressed the growth of Kelly and NGP neuroblastoma xenografts *in vivo* (*p* = 0.036 and *p* = 0.044; Figure 2F) and prolonged the OS of tumor-bearing mice of neuroblastoma xenografts composed of Kelly cells (*p* = 0.0007; Figure 2G). OS was not significantly prolonged in mice bearing NGP-derived neuroblastoma xenografts (Figure S2). No toxicities or weight loss was observed throughout treatment.

### METTL3/14 inhibition promotes adrenergic neuroblastoma cell differentiation

**Figure 3.**
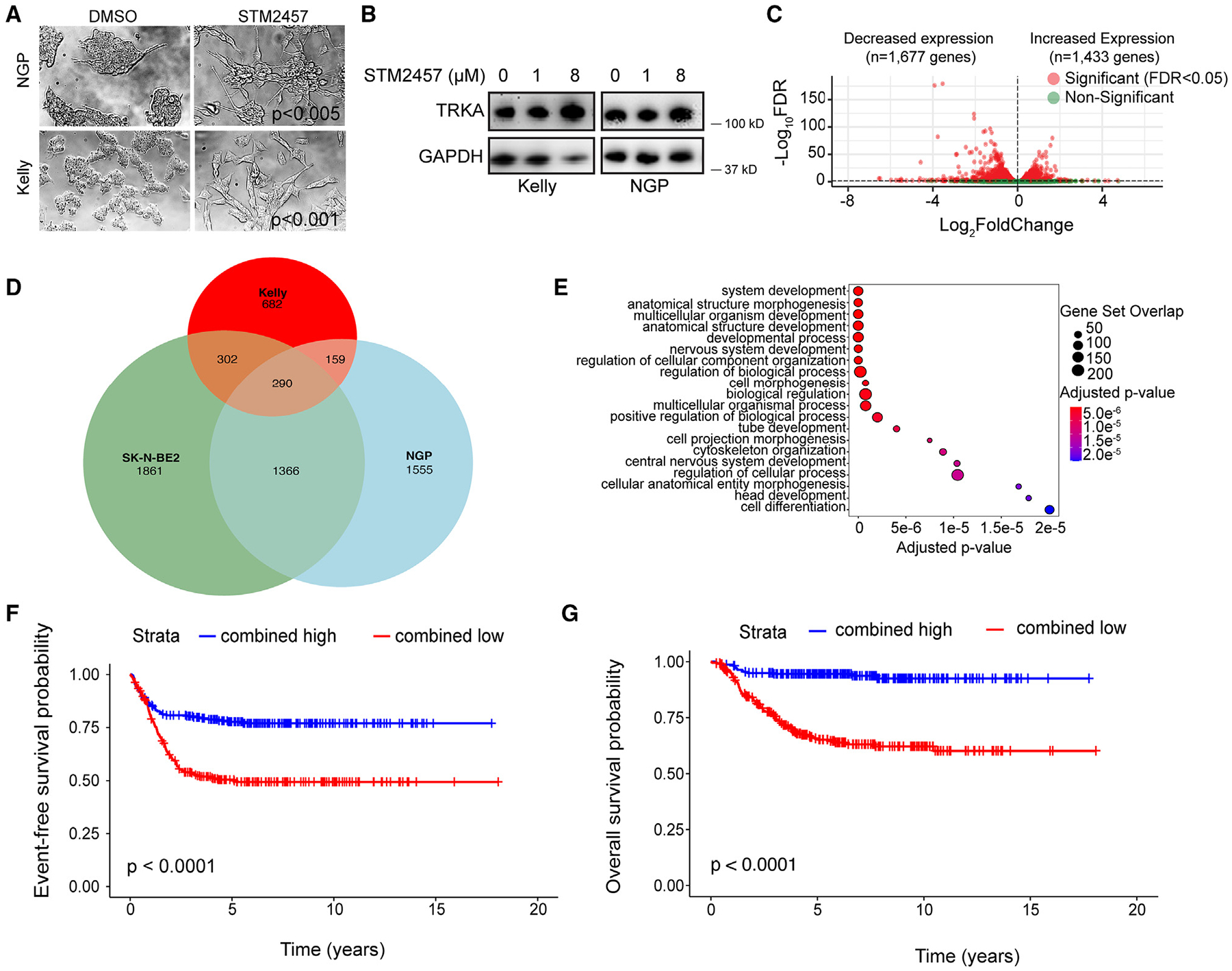
STM2457 treatment promotes neurite outgrowths in neuroblastoma cells and increases expression of neuronal differentiation genes (A) Phase contrast images of Kelly and NGP cells analyzed by the ImageJ processing program show an increase in the length (NGP: *p* < 0.005 and Kelly: *p* < 0.001, unpaired t test) and the number of neurite outgrowths (NGP: *p* < 0.08 and Kelly: *p* < 0.001, unpaired t test) following 6 days of STM2457 treatment (8 μM) compared to control cells treated with DMSO (*n* = 3). (B) TRKA protein expression is increased in Kelly and NGP cells treated for 3 days with 8 μM STM2457. (C) Volcano plot showing differentially expressed genes in Kelly cells treated for 6 days with 8 μM STM2457. (D) Venn diagram showing overlap of up-regulated genes in SK-N-BE2, Kelly, and NGP cells treated with STM2457 for 6 days (12, 8, and 1 μM, respectively). (E) Up-regulated genes that are shared between SK-N-BE2, Kelly, and NGP cells treated with STM2457 for 6 days (12, 8, and 1 μM, respectively) were enriched for GO pathways of nervous system development and cell differentiation. (F and G) Event-free survival (F) and overall survival (G) are significantly improved for patients in the SEQC-NB cohort (*n* = 220) with high (*n* = 220) vs. low (*n* = 273) (*p* < 0.0001 for both, log-rank test) METTL3 inhibitor response signature scores. The signature is comprised of 73 overlapping genes that were up-regulated in NGP, Kelly, and SK-N-BE2 cells following STM2457 treatment and enriched for nervous system development.

Morphologic evidence of differentiation was observed in Kelly and NGP cells following 6 days of STM2457 treatment, with induction of neurite outgrowths, a hallmark of *in vitro* neuroblastoma differentiation, and cell elongation.^22^ The average neurite length in NGP cells increased 1.34 times with STM2457 treatment compared to DMSO (*p* = 0.004; Figure 3A). In Kelly cells treated with the METTL3 inhibitor, the average neurite length increased 1.3 times (*p* = 0.0002). STM2457 treatment also significantly increased the neurite number in Kelly cells compared to DMSO (2.7 times; *p* < 0.0001). A trend associating an increased neurite number with STM2457 treatment compared to DMSO was seen in NGP cells, although the difference was not statistically significant (1.7 times; *p* = 0.08). Western blot analysis demonstrated that STM2457 treatment increased the levels of TRKA, the protein product of *NTRK1*, in Kelly and NGP neuroblastoma cells (Figure 3B). Activation of this neurotrophin receptor by its ligand nerve growth factor initiates a cascade of signaling events leading to neuronal differentiation of TRKA-expressing cells *in vitro*,^23^ and elevated TRKA expression is characteristic of biologically favorable neuroblastoma.^24,25^

METTL3/14 inhibition also led to changes in gene expression in neuroblastoma cell lines. In Kelly cells, the expression levels of 1,433 genes were increased, and 1,677 genes were down-regulated (Figure 3C). In NGP cells, 3,370 genes were up-regulated and 3,444 genes were down-regulated with STM2457 treatment (Figure S3A). In SK-N-BE2 cells, 3,819 genes were up-regulated and 3,812 genes were down-regulated with STM2457 treatment (Figure S3B). In all three cell lines, increased expression of neuronal differentiation genes was observed after 6 days of treatment with the inhibitor, and enrichment of nervous system development and cell differentiation pathways was identified by GOBP analysis (Figures 3D and S3C–S3E). In Kelly cells, changes in gene expression were detected prior to the changes in cell morphology, with enrichment for neuronal differentiation pathways observed after 1 day of STM2457 treatment (Figure S3F). The down-regulated transcripts were enriched for a diverse set of pathways that included cell differentiation and pathways involved in biosynthetic and metabolic processes (Figures S3G–S3J).

In Kelly cells, METTL3/14 inhibition increased the expression of genes associated with differentiated neurons and chromaffin cells^26–28^ (Figure S4; Table S1). The list included genes expressed in chromaffin cells in the mature adrenal gland (*CHGA*, *CHRNA3*), genes that mediate retinoic-acid-induced neuroblastoma differentiation (*SOX4*, *HAND2*, *TBX2*, *TBX3*),^14,29^ and genes that are up-regulated in differentiated neuroblastoma cells (*NTRK1*, *HOXC9*).^24,29,30^ Increased expression scores of a subset of these genes, such as TBX3, CHRNA7, and FN1, was shared among NGP and SK-N-BE2 cells treated with STM2457 (Table S2).

RNA sequencing analysis of neuroblastoma xenografts harvested from mice treated with STM2457 or vehicle demonstrated an alignment rate of 20%–50% with the human genome, reflective of contaminant mouse tissue. Although a high variability in gene expression was detected in the tumor samples, 112 genes were up-regulated in tumors of mice treated with STM2457 compared to controls (adjusted p value [p_adj_] < 0.1, log2 fold change > 0) (Table S3). Increased expression of *CHGA*, *GATA2*, and *TBX2* was observed with STM2457 treatment in the neuroblastoma xenografts.

### A METTL3 inhibitor response signature is prognostic of outcome in neuroblastoma

To investigate the prognostic relevance of genes up-regulated following STM2457 treatment *in vitro*, we first identified a consensus set of 290 genes that were up-regulated in Kelly, NGP, and SK-N-BE2 cells following 6 days of METTL3 inhibitor treatment (Figure 3D). Nervous system development was among the top significantly up-regulated biological pathways in the consensus up-regulated gene set (Figure 3E). Gene set variation analysis was then utilized to develop a gene signature (“METTL3 inhibitor response signature”) based on the subset of the consensus up-regulated gene set that was involved in nervous system development (GO: 0007399) (*n* = 73 genes), and the prognostic value of this gene signature was evaluated in two independent neuroblastoma patient cohorts. Using the SEQC-NB cohort, an optimal signature score cutoff was determined through interrogation of the receiver operator curve and used to categorize tumors as having “high” or “low” expression of the gene subset.^31^ In the SEQC-NB cohort (*n* = 493), high expression of the METTL3 inhibitor response signature correlated with significantly better 5 year EFS (77.65% [95% confidence interval (CI): 72.25%–83.46%] vs. 50.46% [95% CI: 44.69%–56.97%]; *p* < 0.0001) and 5 year OS (94.46% [95% CI: 91.47%–97.56%] vs. 65.15% [95% CI: 59.33%–71.54%]; *p* < 0.0001) compared to patients with neuroblastomas with low expression (Figures 3F and 3G, respectively). Tumors in the Westermann cohort (*n* = 105) were classified as having high or low expression of the METTL3 inhibitor response signature using the same cutoff score identified in the SEQC-NB cohort. In the Westermann cohort (*n* = 105), high expression of the METTL3 inhibitor response signature also correlated with significantly better 5 year EFS (65.41% [95% CI: 53.55%–79.88%] vs. 36.56% [95% CI: 25.21%–53.02%]; *p* = 0.0022) and 5 year OS (88.54% [95% CI: 80.29%–97.63%] vs. 52.70% [95% CI: 39.90%–69.60%]; *p* = 0.00015) comparedto patients with tumors with low signature expression (Figures S5A and S5B, respectively).

### METTL3/14 inhibition reduces m^6^A RNA deposition and increases expression of neuronal differentiation genes

**Figure 4.**
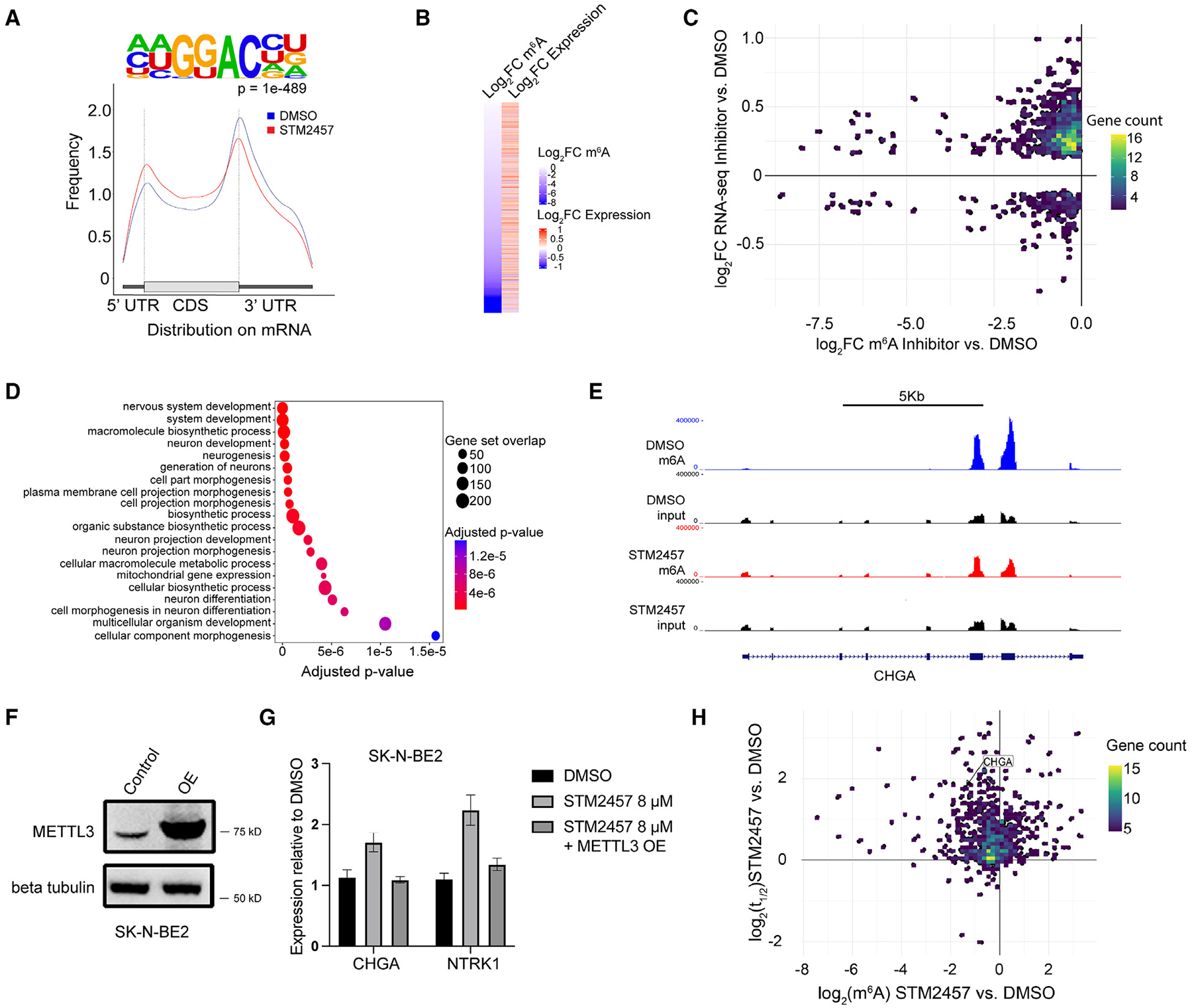
STM2457 treatment decreases m^6^A mRNA deposition in networks enriched for neuronal differentiation and up-regulates expression of transcripts by increasing stability (A) Representative metagene analysis showing m^6^A distribution in control- (blue) and STM2457-treated (red) Kelly and NGP cells and m^6^A located at consensus “DRACH” motif in Kelly cells. (B) Heatmap showing expression value (*Z* scored expression) of genes with m^6^A loss in Kelly cells treated with DMSO or STM2457 (*p* < 0.05, unpaired t test). (C) STM2457 treatment of Kelly cells promotes loss of mRNA m^6^A deposition (x axis) assessed by methylated RNA immunoprecipitation sequencing and increased gene expression assessed by RNA sequencing (y axis) compared to DMSO. (D) GO enrichment analysis of transcripts with decreased m^6^A and increased expression in Kelly cells at 24 h. (E) Tracks of m^6^A enrichment across CHGA in Kelly cells in DMSO- (top) and STM2457-treated cells (bottom). (F) Western blot analysis shows overexpression (OE) of METTL3 in SK-N-BE2 cells transfected with the pkmyc vector cloned with METTL3 compared to control cells. (G) The increase in CHGA and NTRK1 expression assessed by RT-qPCR in SK-N-BE2 cells treated with 16 μM STM2457 is not observed in SK-N-BE2 cells with METTL3 OE (*p* < 0.05, Student’s t test). (H) Half-life of mRNA (y axis) plotted against change in m^6^A level (x axis) in neuroblastoma cells treated with 8 μM STM2457 or DMSO. Bar graphs show mean and SD of data from 3 independent experiments.

STM2457 treatment globally decreased m^6^A mRNA modifications in neuroblastoma cells (Figure 2A). To identify the specific transcripts with m^6^A loss following METTL3 inhibition, methylated RNA immunoprecipitation sequencing was carried out in parallel to RNA sequencing of Kelly and NGP cells. Since m^6^A loss occurs within 24 h of treatment with STM2457, sequencing was carried out at this time point. A representative metagene analysis of Kelly cells treated with STM2457 shows the distribution of m^6^A at the 3′ end, at the 5′ end, and across the gene body of mRNA (Figure 4A). A relative loss of m^6^A is appreciated around the stop codon and 3′ UTR; however, no major changes in m^6^A distribution are present, consistent with prior reports.^5^ As expected, m^6^A was found at the canonical “DRACH” sequence, where D = A, G, or U; R = A or G; and H = A, C, or U.^32^

We next examined the association between m^6^A loss and mRNA expression in neuroblastoma cells treated with STM2457. As shown in Figure 4B, STM2457 treatment for 1 day caused a statistically significant m^6^A loss on 697 transcripts (p_adj_ < 0.05). Expression levels increased in 532 of these genes (log2 fold change > 0, *p* < 0.05) and decreased in 165 genes (log2 fold change < 0, *p* < 0.05) (Figure 4C). After 6 days of treatment with STM2457, m^6^A loss was detected on 294 transcripts (Figure S6A). Expression increased for 218 genes (log2 fold change > 0, *p* < 0.05) and decreased for 76 genes (log2 fold change < 0, *p* < 0.05). GOBP analysis of up-regulated METTL3 targets showed enrichment for genes involved in nervous system development, neuron development, and neurogenesis at 24 h (Figure 4D). After 6 days, GOBP analysis of up-regulated METTL3 targets showed enrichment for genes in pathways of RNA biosynthetic processes and transcription in Kelly cells and of multi-cellular organism development in NGP cells (Figures S6B and S6C, respectively). A representative track of CHGA mRNA with enrichment of m^6^A and input in Kelly cells treated with both DMSO and STM2457 shows loss of m^6^A enrichment near the stop codon and within exon regions with METTL3 inhibitor treatment (Figure 4E). To confirm that the changes in gene expression observed in neuroblastoma cells treated with STM2457 were due to targeting METTL3, we analyzed *CHGA* and *NTRK1* mRNA levels in control SK-N-BE2 cells and cells engineered to overexpress METTL3. Western blot demonstrates that higher levels of METTL3 protein are expressed in SK-N-BE2 cells transfected with the pkmyc vector cloned with METTL3 compared to control cells (Figure 4F). As shown in Figure 4G, METTL3 overexpression suppressed the STM2457 induced up-regulation of *CHGA* (*p* = 0.0027) and *NTRK1* (*p* = 0.0046) observed in control SK-N-BE2 cells, indicating that METTL3 plays a key role in the regulation of these genes.

### METTL3/14 inhibition stabilized target transcripts

We next sought to investigate the mechanisms underlying the increase in expression of targets transcripts with METTL3/14 inhibition observed in neuroblastoma cells. Transcripts marked by m^6^A are often recognized by various reader proteins that bind to and metabolize cognate RNA.^2,33^ The effects of recognition can lead to altered levels of RNA expression by mechanisms such as enhanced stability or increased degradation. Since transcripts marked with m^6^A in control neuroblastoma cells showed increased expression in cells treated with STM2457, we hypothesized that the reduction of m^6^A RNA deposition resulting from METTL3/14 inhibition would enhance the stability of these transcripts. Indeed, over 50% of transcripts with m^6^A loss show increased stability (Figure 4H). Of 6,064 gene transcripts with discernible half-life values across samples and time points, increased half-life (log2 fold change > 0) was detected in 89.2% and 80.5% of the transcripts in the STM2457-treated Kelly and NGP cells, respectively, compared to control cells treated with DMSO. Further, STM2457 treatment increased the the stability of 39.4% of the transcripts in Kelly cells and 57.4% of the transcripts in NGP cells. Genes with decreased m^6^A and increased stability are involved in neurite development, including *KIF26A*, *DOK4*, and *GPRIN1* (Table S4).^34–36^ Other genes involved in the process of differentiation, including *TBX2* and *HOXC9*, were also up-regulated.^29,30^

## DISCUSSION

The m^6^A epitranscriptome plays a critical role in regulating the expression of genes that promote oncogenic growth in many types of adult cancers, although little is known about its function in pediatric cancers.^7^ In this study, we show that high levels of METTL3 and METTL14 in neuroblastoma are associated with aggressive tumor growth and poor survival. *METTL3* knockdown or pharmacologic inhibition of the METTL3/14 complex with STM2457 decreased the proliferation of a panel of adrenergic neuroblastoma cells, including cells that harbored amplification of the *MYCN* oncogene. In addition, STM2457 suppressed the growth of *MYCN*-amplified neuroblastoma xenografts and prolonged the survival of tumor-bearing mice. STM2457 treatment induced morphologic changes that are characteristic of *in vitro* neuroblastoma differentiation and led to up-regulation of neuronal differentiation genes. Further, METTL3 overexpression suppressed the induction of neuronal differentiation gene expression observed with STM2457 treatment, indicating that the transcriptomic changes were due to on-target effects. METTL3/14 inhibition reduced m^6^A deposition on target transcripts, leading to stabilization and increased expression of mRNAs critical for sympathoadrenal development, cell identity, and neuronal differentiation. We also show that the changes in gene expression mediated by the METTL3 inhibitor are prognostic of survival in patients with neuroblastoma. In two clinically annotated neuroblastoma cohorts (SEQC-NB [*n* = 498] and Westermann [*n* = 105]), superior EFS and OS were observed among patients with tumors with high METTL3 inhibitor response signature scores compared to those with low scores. These results suggest that the genes regulated by METTL3 inhibition influence the clinical behavior of neuroblastoma tumors.

Up-regulated METTL3 drives oncogenic tumor growth in many types of adult malignancies including lung, breast, and colon cancers.^4,20,37^ In AML, METTL3 increases mRNA stability and translation of m^6^A-marked oncogenes such as MYC.^38–40^ However, in other neoplasms, including endometrial cancer, decreased m^6^A levels are associated with tumor growth. In hepatocellular carcinoma and glioblastoma, the role of METTL3 is unclear due to conflicting findings about whether the m^6^A methyltransferase complex promotes or inhibits cancer pathogenesis.^8,41,42^

METTL3-mediated m^6^A RNA modifications are known to play an important role in embryonic stem cell development and postnatal hematopoietic and neural stem cell self-renewal.^43–45^ m^6^A marks a wide range of transcripts between stages of development and can be used to passage cells through stages of differentiation.^1,2,46–48^ mRNA modifications also contribute to the regulation of tumor cell fate, and m^6^A modifications are commandeered to maintain primitive, proliferative states in many types of cancer cells.^38,39^ In AML, METTL3 depletion down-regulated cell cycle pathway genes and up-regulated hematopoietic cell differentiation genes.^38^ More recently, Yankova and colleagues demonstrated that METTL3 inhibition with STM2457, a small-molecule inhibitor first identified and characterized by these investigators, reduced AML growth, increased cell differentiation, and impaired engraftment in various mouse models.^5^

We show that STM2457 also suppressed neuroblastoma growth and promoted cell differentiation. METTL3/14 inhibition reduced m^6^A RNA deposition and increased the stability and expression of target transcripts. Up-regulated genes included *PHOX2B*, *SOX4*, and *KIF26A*, which encode transcription factors in the gene regulatory network that controls initial sympathetic neuron development and are essential for the maintenance of differentiated neurons.^34,49^ The METTL3 inhibitor also increased the expression of *TBX2* and *TBX3*, genes that encode transcription factors involved in the patterning, specification, proliferation, and differentiation programs in vertebrate organogenesis.^26–28,50,51^ In addition, the expression of differentiation markers including *TUBB3*, *HOXC9*, and *NTRK1* was up-regulated in neuroblastoma cells treated with STM2457.^24,29,30^ Once placed, m^6^A is detected by reader proteins that bind to and direct the transcript for a multitude of functions.^52^ In the cytoplasm, this typically includes mediating mRNA stability, mRNA degradation, and translation of mRNA into protein. It is likely that binding of a reader protein dictates the increased expression seen in our study.

Collectively, our results show that m^6^A RNA modifications play a critical role in regulating the expression of METTL3-target transcripts, neuroblastoma phenotype, and tumor growth. METTL3 inhibitor treatment suppressed neuroblastoma growth and increased differentiation, supporting the potential of this therapeutic strategy for neuroblastoma. Future studies evaluating more potent, clinical-grade inhibitors of METTL3 are warranted.

### Limitations of the study

Although the results of our study support the potential of *METTL3* as a therapeutic target in neuroblastoma, there are limitations. METTL3 protein was not completely knocked down in neuroblastoma cells by either of the two METTL3 siRNAs tested, and only modest reductions in global m^6^A levels were observed in the METTL3 knockdown neuroblastoma cells. It is possible that genetic knockout of METTL3 using CRISPR technology may promote neuroblastoma differentiation and other alterations in the cell phenotype that more closely mirror the changes observed in cells treated with the METTL3 inhibitor. Another limitation is that the effects of pharmacologic inhibition of METTL3/14 were analyzed in neuroblastoma cell lines and subcutaneous neuroblastoma xenograft models. Although it is possible that the antitumor activity of METTL3/14 inhibition may differ in ortho-topic models, patient-derived xenograft tumors, or other preclinical neuroblastoma models, it is well recognized that all preclinical models have inherent limitations and are not consistently predictive of response in patients. Despite these limitations, our studies support the potential for METTL3/14 inhibition as a therapeutic strategy for neuroblastoma.

## STAR⋆METHODS

### RESOURCE AVAILABILITY

#### Lead contact

Further information and requests for resources and reagents should be directed to and will be fulfilled by the lead contact, Susan L. Cohn (scohn@bsd.uchicago.edu).

#### Materials availability

Methods to synthesize STM2457 generated in this study are described in the STAR Methods section.

#### Data and code availability

- RNA sequencing, publicly available expression data, survival data, and meRIP sequencing have been deposited at GEO and are publicly available as of the date of publication. Accession numbers are listed in the key resources table. Microscopy data reported in this paper will be shared by the lead contact upon request.
- This paper does not report original code.
- Any additional information required to reanalyze the data reported in this paper is available from the lead contact upon request.

### EXPERIMENTAL MODEL AND STUDY PARTICIPANT DETAILS

#### Cell culture

Neuroblastoma cell lines Kelly, NGP, SH-SY5Y, SK-N-AS, SHEP, NBL-W-N, NBL-W-S, LAN-5, LA1–55n, LA1–5s, and SK-N-BE2 were grown at 37° C with 5% CO_2_ in RPMI 1640 (Gibco, 11875–093) supplemented with 10% heat-inactivated FBS (Gibco, 26140–079), 2 mmol/L L-glutamine (Gibco, 25030–081), and 1% Antibiotic-Antimycotic (Gibco, 15240–062). NBL-W-N and NBL-W-S were established in our laboratory,^55,56^ SHEP, SH-SY5Y, and SK-N-BE2 were kind gifts from Dr. June Biedler (Memorial Sloan Kettering), Kelly and NGP were obtained from ATCC, SK-N-AS was a kind gift from Dr. John Maris, LA1–55n, LA1–5s, and LAN-5 were kind gifts from Dr. Robert Seeger. All cell lines tested negative for *Mycoplasma* contamination using the MycoAlert Detection Assay (Lonza, LT07–705).

#### *In vivo* animal studies

Athymic nude mice 4–6 weeks of age, of equal ratio male and female were used for xenograft studies and were purchased from Harlan. Mice were maintained in the Animal Facility at University of Chicago. All experiments were performed in accordance with national guidelines and regulations and approved by the University of Chicago Institutional Animal Care and Use Committee (Chicago, IL) under protocol #71829. For each *in vivo* experiment, equal numbers (10 each) of male and female nude mice were subcutaneously injected with 1 × 10^7^ Kelly or 1 × 10^6^ NGP neuroblastoma cells suspended in 200μL containing Cultrex basement membrane extract per manufacturer’s instructions (R&D Systems, 3632-005-02). Animals were randomized to treatment or control groups when tumors were palpable, at approximately 70 mm^3^. All animals were sacrificed when tumors reached a terminal size of 1.5 cm^3^.

### METHOD DETAILS

#### Knockdown and overexpression experiments

Kelly cells were incubated with siRNA against METTL3 (Integrated DNA Technologies, 300621825 and 300621828) or negative control siRNA (Integrated DNA Technologies, 51-01-19-09). siRNA was transiently transfected into Kelly cells using lipofectamine RNAiMax (Invitrogen, 13778075) per manufacturer’s instructions. Cells were incubated with 20 μM siRNA in 6 well plates for 72 h and collected for analysis. For overexpression studies, METTL3 (Liu et al.,^18^ 53739) were cloned into pkmyc (Joberty et al.,^57^ 19400) then transfected into SK-N-BE2 cells by Lipofectamine 2000 using manufacturer’s instructions.^69^ Briefly, cells were seeded 24 h before transfection, and the plasmid(s) and Lipofectamine reagent was combined in Opti-MEM media. Media with Lipofectamine 2000 was then replaced with media only without antibiotics after 6 h. After 2 days, cells were treated with DMSO or 16 μM STM2457 for 3 days (qPCR for *CHGA* and *NTRK1*) or 6 days (detection of cleaved PARP by western blot), or for 2, 4, and 6 days for growth assessment.

#### Synthesis of STM2457

STM2457 was synthesized with reference to the structure published previously (Figure S7).^5^ A flask was charged with 2-aminopyridine (1, 6.022g, 64 mmol) and water (1.2 L). Then dimethyl acetylenedicarboxylate (2, 10.800g, 76 mmol) was added to the reaction system. The reaction was stirred at room temperature for 24 h with reaction system exposed in air. The mixture was then extracted with CH2Cl2 (500 mL) 3 times, and the combined organic layers were washed with brine and dried over anhydrous Na2SO4, filtered, and concentrated. The residue was purified by silica gel column chromatography (eluting with 1:1:1 hexanes/ethyl acetate/acetone) to afford compound 3 (8.015 g, 62%.) as a light-yellow solid. A suspension of methyl 4-oxopyrido[1,2-*a*]pyrimidine-2-carboxylate (3, 3.014 g, 14.76 mmol) in 8M HCl (7.5 mL) solution was made that became clear after heating for 10 min. The reaction was heated for an additional 1 h and the clear reaction system returned to the suspension. The mixture was cooled to room temperature and the precipitate was collected by filtration and dried under vacuum to yield compound 4 (2.68 g, 96%%) as a white solid. To a stirred solution of methyl 6-aminonicotinate (5, 4.0 g, 26.28 mmol) in acetonitrile (40 mL) 1, 3-dichloropropan-2-one (6, 6.673 g, 52.56 mmol) was added at room temperature under a nitrogen atmosphere and the resulting reaction mixture was heated at 80° C for 16 h. After the starting materials were consumed, the mixture was cooled to room temperature, diluted with saturated aqueous sodium hydrogen carbonate (50 mL) and extracted with ethyl acetate (100 mL) 3 times. The combined organic layers were washed with brine, dried over anhydrous sodium sulfate, and concentrated in vacuo. The crude product was purified by flash column chromatography (eluting with 1:1 hexanes/ethyl acetate) to afford product 7 (4.5 g, 77%) as a white foam. A solution of methyl 2-(chloromethyl)imidazo[1,2-*a*]pyridine-6-carboxylate (7, 1.400 g, 6.24 mmol) in anhydrous THF (20 mL) was cooled in an ice/water bath. DIBAL (1M in toluene, 17.2 mL, 17.2 mmol) was added slowly to yield a yellow solution and the mixture stirred and cooled for 2 h. The reaction mixture was quenched by dropwise addition of methanol (10 mL), then diluted with dichloromethane (100 mL) and saturated aqueous sodium hydrogen carbonate (50 mL). The mixture was filtered through Celite and the residue wash with dichloromethane. The filtrates were phase-separated, the aqueous layer extracted with dichloromethane (50 mL) 3 times. The combined organic layers were washed with brine, dried over anhydrous sodium sulfate, and concentrated in vacuo. The crude product was purified by flash column chromatography (eluting with 1:1 hexanes/acetone) to afford product 8 (780 mg, 64%) as a yellow foam. To a stirred solution of [2-(chloromethyl)imidazo[1,2-*a*]pyridin-6-yl]methanol (8, 510 mg, 2.602 mmol), sodium iodide (39 mg, 0.260 mmol) and sodium azide (400 mg, 6.154 mmol) were combined in dimethylformamide (3 mL) and the mixture stirred at room temperature for 3 h. The reaction mixture was diluted with saturated aqueous sodium hydrogen carbonate (30 mL) and extracted with ethyl acetate (50 mL) 3 times. The combined organic layers were washed with brine, dried over anhydrous sodium sulfate, and concentrated in vacuo. The crude product was purified by flash column chromatography (eluting with 1:1 hexanes/acetone) to yield product 9 (500 mg, 95%) as a white solid. A stirred solution of [2-(azidomethyl)imidazo[1,2-*a*]pyridin-6-yl]methanol (9, 710 mg, 3.49 mmol) in dichloromethane (20 mL) was cooled in an ice/water bath. Dess-Martin periodinane (1.925 g, 4.540 mmol) was added slowly to the suspension reaction system. The mixture was then naturally warmed to room temperature and stirred at room temperature overnight. The reaction mixture was diluted with saturated aqueous sodium hydrogen carbonate (10 mL) and extracted with ethyl acetate (50 mL) 3 times. The combined organic layers were washed with brine, dried over anhydrous sodium sulfate, and concentrated in vacuo. The crude product was purified by flash column chromatography (eluting with 2:1 hexanes/acetone) to afford product 10 (700 mg, quant.) as a white solid. To a stirred solution of compound 10 (480 mg, 2.385 mmol), starting material 11 (540 mg, 4.772 mmol) was combined in hexafluoro-2-propanol (HFIPA, 10 mL) and the mixture stirred at room temperature for 30 min. Another part of starting material 11 (540 mg, 4.772 mmol) was added and the mixture stirred at room temperature for 1.5 h. The sodium borohydride (81.2 mg, 2.147 mmol) and a few drops of methanol were added to the reaction system. After the reaction completed, the reaction mixture evaporated under vacuum and residue was suspended in saturated aqueous sodium hydrogen carbonate (10 mL) and extracted with chloroform/isopropyl alcohol (3:1, 40 mL) 3 times. The combined organic layers were dried over anhydrous sodium sulfate and concentrated in vacuo. The crude product was purified by flash column chromatography (eluting with 10:1 dichloromethane/methanol) to yield product 12 (600 mg, 85%) as a colorless liquid. A solution of compound 12 (600 mg, 2.011 mmol) in methanol (20 mL) was stirred at room temperature. Di-*tert*-butyl decarbonate (879 mg, 4.027 mmol) was added slowly to yield a clear reaction system. After stirring at room temperature for 16 h, another part of Di-*tert*-butyl decarbonate (879 mg, 4.027 mmol) was added and the mixture stirred at room temperature for an additional 2 h. Then the reaction mixture evaporated under vacuum and residue was suspended in saturated aqueous sodium hydrogen carbonate (10 mL) and extracted with ethyl acetate (50 mL) 3 times. The combined organic layers were dried over anhydrous sodium sulfate and concentrated in vacuo. The crude product was purified by flash column chromatography (eluting with 20:1 dichloromethane/methanol) to yield product 13 (800mg, quant.) as a white solid. A solution of compound 13 (1.183 g, 3.121 mmol) in methanol (30 mL) was stirred at room temperature. Pd/C (250 mg, 20% w/w) was added to give black suspension reaction system. An H2 (g) balloon was added to the reaction system. Then the mixture was stirred at room temperature for 1 h. Thin-layer chromatography detected the reaction system and the starting materials were consumed. The mixture was filtered through Celite and the residue washed with methanol. The filtrate was concentrated in vacuo and the crude product was used directly in the next step without purification.

A solution of reduced crude product (about 3.121 mmol) in dimethylformamide (20 mL) was stirred at room temperature. Hexafluorophosphate azabenzotriazole tetramethyl uronium (HATU, 1.780 g, 4.682 mmol) was added slowly to yield a suspension reaction system. Then the *N,N*-diisopropylethylamine (2.017g, 15.605 mmol) was added to the reaction system and stirred at room temperature overnight. The reaction mixture was diluted with saturated aqueous sodium hydrogen carbonate (10 mL) and extracted with ethyl acetate (50 mL) 3 times. The combined organic layers were washed with brine, dried over anhydrous sodium sulfate, and concentrated in vacuo. The crude product was purified by flash column chromatography (eluting with 10:1 dichloromethane/methanol) to afford product 14 (1.012 g, 60% two steps) as a purple foam. To synthesize STM2457, a solution of compound 14 (500 mg, 0.918 mmol) in dichloromethane (20 mL) was stirred at room temperature. Trifluoroacetic acid (10 mL) was added to the reaction system. Then the mixture was stirred at room temperature for 2 h. Thin-layer chromatography detected the reaction system, and the starting material was consumed. The reaction mixture was concentrated in vacuo and crude product obtained. The combined organic layers were washed with brine, dried over anhydrous sodium sulfate, and concentrated in vacuo. The crude product was purified by flash column chromatography (eluting with 10:1 to 8:1 dichloromethane/methanol) with a short column to yield product STM2457 as a yellow foam. The pure yellow foam product was dissolved in acetonitrile-water (1:1, ca. 50 mL) and freeze dried to give STM2457 (356 mg, 88%) as a white solid.

#### Western blot analysis

Protein lysates of METTL3 knockdown neuroblastoma cells and cells with METTL3 inhibition by STM2457 were prepared by boiling cell pellets in buffer containing 50 mM Tris-HCl pH 6.8, 2% SDS and protease inhibitor (Sigma, PB340) and phosphatase inhibitor (Sigma, 124K4131) cocktail for 5 min. Protein concentrations were determined with the BCA Protein Assay Reagent (Pierce, 23225). 30 μg of total protein were electrophoresed on Stain Free 4–20% SDS-PAGE gradient gels (BioRad) and transferred to nitrocellulose membranes (Amersham, 10600002). Gel activation images for total protein amounts were obtained according to BioRad ChemiDoc XRS+ protocol. Membranes were blocked in Tris-Buffered Saline (TBS, pH 7.4) with 0.1% Tween 20 and 5% nonfat dry milk. Antibodies for MYCN (Cell Signaling, 9405s), METTL3 (Abcam, 195352), METTL14 (Sigma, HPA038002), Myc-Tag (Cell Signaling Technologies, 2276), PARP (Cell Signaling Technology, 9532), and TRKA (Abcam, 302524) were used at a 1:1000 dilution. Appropriate secondary antibodies were used and blots were developed with Clarity Western ECL Substrate (BioRad, 1705061). Images were acquired on a BioRad ChemiDoc XRS+ imaging system and analyzed using Image Lab software.

#### Cell morphology

NGP and Kelly cells were plated in chamber slides and treated with either 8 μM STM2457 or DMSO for 6 days. The cells were fixed with 4% PFA for 45 min and stored in PBS at 4°C until imaging. A Nikon Eclipse Ti inverted microscope at 20X magnification was used to generate phase contrast images. Neurite outgrowth was quantified on images of control or STM2457-treated cells using the ImageJ processing program. Three separate experiments were evaluated, and the average neurite length and neurite number were calculated for each cell line and treatment condition with the ImageJ software (Rasband, W.S., ImageJ, U. S. National Institutes of Health, Bethesda, Maryland, USA, https://imagej.net/ij/, 1997–2018) running NeuronJ plugin.^70^

#### Cell viability

The effects of siRNA and STM2457 on neuroblastoma cell proliferation were analyzed using the CellTiter 96 Aqueous Non-radioactive Proliferation Assay Kit (Promega, G9241). For experiments with STM2457, cells were incubated in a 96 well plate for six days in growth media with concentrations of drug at concentrations ranging from 0 to 400 μM. After incubation, 3-(4,5-dimethylthiazol-2-yl)-5-(3-carboxymethoxyphenyl)-2-(4-sulfophenyl)-2H-tetrazolium (MTS) was added and absorbance measured using a Synergy 2 Microplate Reader (Bio-Tek Instruments). A nonlinear regression, sigmoidal four-parameter dose–response model was used to determine IC_50_ by plotting log of inhibitor concentration versus relative survival using Prism software (GraphPad Prism 9). For over-expression studies, cell viability was assessed using sulforhodamine B (SRB) colorimetric assay.^71^ After treatment, cells were fixed with 10% weight per volume TCA, then stained with 0.05% SRB. After washing 3–4 min with water, bound SRB was solubilized with 10mM Trizma base and measured at 515nm.

#### Quantitative analysis of m^6^A levels

mRNA was isolated using Dynabeads mRNA DIRECT Purification kit (Invitrogen, 61012). 50 ng mRNA was digested by nuclease P1 (Sigma, N8630) in 15 μL of buffer containing 0.3 μL 1 M NH_4_Ac overnight at 37° C. Subsequently, 1 unit of FastAP (Thermo Scientific, EF0651) in 10x FastAP buffer was added and the sample was incubated for 2 h at 37° C. The samples were then filtered (0.22 μm, Millipore) and injected into a C18 reverse-phase column coupled to an Agilent 6460 LC-MS/MS spectrometer. The nucleosides were quantified using retention time and the nucleoside to base ion mass transitions (268–136 for A; 282 to 150 for m^6^A). Quantification was performed by comparison to the standard curve obtained from pure nucleoside standards running with the same batch of samples. The m^6^A level was calculated as the ratio of m^6^A to A.

#### Cell-cycle analysis

Kelly cells were treated with STM2457 from 0 to 16 μM for 6 days, harvested and washed with cold PBS (pH 7.4) followed by Versene at 37° C for 5 min. The cells were pelleted at 1,500 rpm for 3 min, washed with PBS, and 70% ethanol was added dropwise while vortexing, followed by incubation at −20° C. Before cytometric analysis, cells were washed twice with PBS and 1 × 10^6^ cells were filtered and incubated in 0.5 mL of Propidium Iodide/RNase Staining Buffer (BD Biosciences) for 15 min. Cells were then resuspended in a final PBS solution. Cellular DNA content was determined using a BD LSR II flow cytometer using BD FACSDiva Software (BD Biosciences) and analyzed using FlowJo v10.8 Software (BD Life Sciences).

#### Xenograft studies

STM2457 was dissolved in 20% weight per volume 2-hydroxypropyl β-cyclodextrin (Sigma, H107).^5^ The treatment group received 50 mg/kg STM2457 and control animals received vehicle alone. STM2457 or vehicle were injected intraperitoneally once daily for 14 days. Tumor size was measured using a caliper and mice were weighed at time of measurement. Tumors were resected and parts of the tissue were snap-frozen for RNA-seq or fixed in formaldehyde and paraffin-embedded for pathologic evaluation.

#### Gene expression and survival analysis in neuroblastoma

Expression data and associated phenotypic data for 498 neuroblastoma tumors (SEQC-NB cohort) and a distinct set of 105 neuroblastoma tumors (Westermann) were downloaded from the R2 database (r2.amc.nl).^53,54^ In the SEQC-NB cohort, 493 tumors had known *MYCN* amplification status and were included in downstream analyses. The *GSVA* package (v1.46.0) was utilized to perform gene set variation analysis (GSVA).^58^ Significantly up-regulated overlapping genes were identified in Kelly, NGP, and SK-N-BE2 cells following 6 days of treatment with 8 μM, 1 μM, and 12 μM STM2457, respectively.^58^ GOBP analysis identified a subset of the shared genes (*n* = 73) enriched for nervous system development (GO:0007399). Signature scores based on the expression of the 73 gene subset were calculated using GSVA. Through ROC analysis in the SEQC-NB cohort, an optimal score cutoff of 0.01 was identified and used to categorize tumors as METTL3 inhibitor response “high” or “low.” The prognostic value of METTL3 inhibitor response signature scores were investigated in the SEQC-NB and the Westermann neuroblastoma cohort. The same cutoff score was utilized in the SEQC-NB and Westermann cohorts. AUROC analyses of the METTL3 inhibitor response signature scores for EFS and OS were implemented using the pROC package (v1.18.0).^31^ Kaplan-Meier analysis was utilized to determine EFS and OS.^72^ Differences in survival between biologic subsets were assessed using the log rank test. A *p*-value less than 0.05 on the log rank test was considered significant. All survival analyses were performed using the “survival” package (v3.5–3).^73^

GSVA was also utilized to generate a METTL3/14 signature based on the expression of METTL3 and METTL14. The optimal cutoff for the METTL3/14 signature score to categorize tumors as METTL3/14 “high” or “low” was determined to be 0.072 through interrogation of the ROC curve. The prognostic value of the METTL3/14 signature score was investigated in the SEQC-NB and the Westermann neuroblastoma cohort using AUROC analyses for EFS and OS implemented using the pROC package (v1.18.0).^31^

#### Poly(A) mRNA selection and RNA-Sequencing

Total RNA was harvested from cells treated with STM2457 or DMSO for six days using TRIzol reagent (Invitrogen, 15596026). Poly(A) RNA was extracted using the Dynabeads mRNA DIRECT Purification kit (Invitrogen) following the manufacturer’s protocol. Raw read quality for all sequencing data was assessed using fastqc (version 0.11.5) with default settings.^74^ For RNA-Seq data, Trimmomatic (version 0.36) was used to trim leading and trailing bases below quality 3 and remove reads less than 36 base pairs in length.^61^ Reads were aligned to GRCh38 using Hisat2 (version 2.1.0).^62^ Reads were counted across exons (-exon flag) using featureCounts of subread (version 1.5.3).^63^ Raw counts were loaded into DeSeq2 (version 1.38.3) for differential expression analysis.^64^ Adjusted *p*-value of 0.05 was considered significant. gProfiler2 (version 0.2.1) was utilized for gene set enrichment analysis.^59^

#### RT-qPCR

RNA was isolated using TRIzol reagent and concentration was determined using UV spectroscopy (DeNovix). Reverse transcription was performed using Superscript III (Life Technologies, 18080093) according to the manufacturer’s instructions. Real time-quantitative polymerase chain reactions (RT-qPCR) reactions were set up in a 96-well format with 1 × Power SYBR Green Master Mix (Applied Biosystems) and 250 nmol/L forward and reverse primers in 20 μL in a 96-well format. Real-time fluorescence detection of PCR products was performed in a 7500 Fast Real-Time PCR System (Applied Biosystems) with 1 cycle at 95° C for 10 min, 40 cycles at 95° C for 15 s, and 60° C for 1 min. Relative quantities of mRNA expression were determined using the comparative ΔΔC_t_ method. Primer sequences are listed in Table S5.

#### Methylated RNA immunoprecipitation sequencing (MeRIP-seq)

1 μg of poly(A) RNA was used for each sample. m^6^A and non-m^6^A spikein RNA from the EpiMark N6-Methyladenosine Enrichment Kit (NEB, E1610S) were added as the normalization controls for m^6^A level analysis. RNA was diluted to 100 μL and fragmented by 30 cycles of sonication. 1 μg of m^6^A antibody from the same kit was incubated with 25 μL of protein G beads at 4° C for 30 min with rotation. Beads were then washed with reaction buffer (150 mM NaCl, 10 mM Tris-HCl, 0.1% NP-40), rotated with RNA samples at 4° C for 1 h, washed with reaction buffer, low salt wash buffer (50 mM NaCl, 10 mM Tris-HCl, 0.1% NP-40), high salt wash buffer (500 mM NaCl, 10 mM Tris-HCl, 0.1% NP-40), and eluted with 100 μL of RLT buffer (Qiagen, 79216). Enriched RNA was purified by RNA Clean & Concentrator Kits (Zymo Research, R1016) and cDNA libraries were constructed using the SMART cDNA Library Construction Kit (Takara, 634901) following the manufacturer’s instructions. Extracted RNA was sequenced at the Genomics Facility at the University of Chicago.

#### meRIP seq differential methylation analysis

For meRIP-Seq data, Trimmomatic (version 0.36) was used to trim leading and trailing bases below quality 3 and remove reads less than 36 base pairs in length. Reads were aligned to GRCh38 using Hisat2 (version 2.1.0). Bam files were analyzed using MeRIPTools (version 0.2.1) and RADAR (version 0.2.4)^65,66^. m^6^A peaks were called using the callPeakFisher function with minimum counts of 15, FDR cutoff of 0.05, odds ratio cutoff of 1, and joint peak threshold of 3 samples. Differential methylation analysis was performed using the “QNBtest” function in MeRIPTools and the RADAR method with an FDR cutoff of 0.1 and beta cutoff of 0.5. Metagene plots were plotted using the plotMetaGene function in MeRIPTools. deepTools (version 2.0) was utilized to generate bigwig files normalized to reads per kilobase million (RPKM).^67^ Bigwig files were visualized using UCSC Genome Browser.^60^

#### mRNA decay analysis

Cells were treated with 8 μM STM2457 or DMSO for six days to about 80% confluency, rinsed with 1x PBS (Gibco) and treated with Actinomycin D (Sigma, A1410) at a concentration of 10 μg/mL for various time points. Total RNA was harvested using TRIzol. 2 ng of total RNA was used for each cDNA library and 1 μL of ERCC RNA Spike-In Mix (Invitrogen) was added to each sample as the normalization control. cDNA libraries were constructed using the SMART cDNA Library Construction Kit following the manufacturer’s instructions. Sequencing was performed at the Genomics Facility at the University of Chicago. Trimmomatic (version 0.36) was used to trim leading and trailing bases below quality 3 and remove reads less than 36 base pairs in length. Reads were aligned to GRCh38 along with ERCC RNA spike-in control (Thermo Fisher Scientific, 4456740) using Hisat2 (version 2.1.0). Reads were counted across exons (-exon flag) and ERCC RNA Spike-in using a custom GTF file with featureCounts of subread (version 1.5.3). Raw reads were normalized to counts per million (CPM) using edgeR (3.40.2) and converted to atommoles through linear fitting of the ERCC RNA Spike-in controls. mRNA half-life was calculated according to the methodology described in a prior study.^75^ Briefly, half-life was estimated with an assumption of first order decay, where the change in RNA concentration is directly proportional to the constant of RNA decay (K_decay_) and the RNA concentration. With this assumption, half-life (t_1/2_) = ln(2)/K_decay_.

### QUANTIFICATION AND STATISTICAL ANALYSIS

All *in vitro* experiments were repeated at least three times in triplicate and data are presented as mean ± SD unless otherwise noted. The number of replicates for each experiment (number of independent experiments for *in vitro* cell-based assays, number of mice for *in vivo* animal studies) is indicated in the figure legends. All quantitative values obtained in the experiments were evaluated using unpaired Student *t* test and Log rank (Mantel-Cox) tests. A *p*-value of 0.05 was required to ascertain statistical significance. Statistical analysis was carried out using GraphPad Prism 9 or R program.^76^

## Supplementary Material

1

2

## Figures and Tables

**Table:** KEY RESOURCES TABLE

REAGENT or RESOURCE	SOURCE	IDENTIFIER
Antibodies		
MYCN	Cell Signaling Technology	CAT# 9405s; RRID: AB_10692664
METTL3	Abcam	CAT# 195352; RRID: AB_2721254
METTL14	Sigma	CAT# HPA038002; RRID: AB_10672401
TRKA	Abcam	CAT# 302524
Beta-Tubulin	Cell Signaling Technology	CAT# 2146; RRID: AB_2210545
PARP (46D11)	Cell Signaling Technology	CAT# 9532; RRID: 659884
Myc-tag (9B11)	Cell Signaling Technology	CAT# 2276; RRID: 331783
GAPDH (14C10)	Cell Signaling Technology	CAT# 2118; RRID: 561053
Chemicals, peptides, and recombinant proteins		
STM2457	This paper, Yankova et al.^5^	N/A
RPMI 1640	Gibco	11875-093
FBS	Gibco	26140-079
l-glutamine	Gibco	25030-081
Antibiotic-Antimycotic	Gibco	15240-062
Lipofectamine RNAiMax	Invitrogen	13778075
Lipofectamine 2000 Transfection Reagent	Thermo Fisher	11668027
Protease Inhibitor	Sigma	PB340
Phosphatase Inhibitor	Sigma	124K4131
BCA Protein Assay Reagent	Pierce	23225
Nitrocellulose membranes	Amersham	10600002
Clarity Western ECL Substrate	BioRad	1705061
Nuclease P1	Sigma	N8630
FastAP	Thermo Fisher Scientific	EF0651
Cultrex basement membrane	R&D Systems	3632-005-02
2-hydroxypropyl β-cyclodextrin	Sigma	H107
TRIzol	Invitrogen	15596026
Superscript III	Life Technologies	18080093
Spikein RNA	New England Biolabs	E1610S
RLT Buffer	Qiagen	79216
Actinomycin D	Sigma	A1410
ERCC RNA spike-in control	Thermo Fisher Scientific	4456740
SRB	Fluka	86183
Critical commercial assays		
CellTiter 96 Aqueous Non-radioactive Proliferation Assay Kit	Promega	G9241
Dynabeads mRNA DIRECT Purification kit	Invitrogen	61012
RNA Clean & Concentrator Kit	Zymo Research	R1016
SMART^®^ cDNA Library Construction Kit	Takara	634901
MycoAlert Detection Assay	Lonza	LT07-705
Deposited data		
Raw and analyzed data	This paper	GEO: GSE240595
SEQC-NB patient cohort	R2 database^53^	GEO: GSE49710
Westermann patient cohort	R2 database^54^	GEO: GSE73518
Experimental models: Cell lines		
Human: Kelly	ATCC	N/A
Human: NGP	ATCC	N/A
Human: SH-SY5Y	Laboratory of June Biedler	N/A
Human: SK-N-AS	Laboratory of John Maris	N/A
Human: SHEP	Laboratory of June Biedler	N/A
Human: NBL-W-N	Our laboratory^55,56^	N/A
Human: NBL-W-S	Our laboratory^55,56^	N/A
Human: LAN-5	Laboratory of Robert Seeger	N/A
Human: LA1-55n	Laboratory of Robert Seeger	N/A
Human: LA1-5s	Laboratory of Robert Seeger	N/A
Human: SK-N-BE2	Laboratory of June Biedler	N/A
Experimental models: Organisms/strains		
Nude mice	Harlan	N/A
Oligonucleotides		
siRNA targeting METTL3 #1	Integrated DNA Technologies	30062182
siRNA targeting METTL3 #2	Integrated DNA Technologies	300621828
Negative Control siRNA	Integrated DNA Technologies	51-01-19-09
Recombinant DNA		
pcDNA3/Flag-METTL3	Liuetal.^18^	53739
pKMyc	Joberty et al.^57^	19400
Software and algorithms		
Image Lab Software	Bio-Rad	https://www.bio-rad.com/en-us/product/image-lab-software?ID=KRE6P5E8Z&WT.mc_id=170519018950&WT.srch=1&WT.knsh_id=cr189944&s_kwcid=AL!18120!3!657783331339!e!!g!!image%20lab%20software&gclid=Cj0KCQjwib2mBhDWARIsAPZUn_m5lc64rSNM-Q9SY5cEVPhie0cjYiDHwJ4EuqHFtUEbKW8w3Dpgi5IaAmKKEALw_wcB
GraphPad Prism 9	GraphPad	https://www.graphpad.com
BD FACSDiva Software	BD Biosciences	https://www.bdbiosciences.com/en-us/products/software/instrument-software/bd-facsdiva-software
FlowJov10.8	Flowjo, LLC	https://www.flowjo.com
R Program	R Core Team	https://www.r-project.org/
*GSVA* package (version 1.46.0)	Hanzelman et al.^58^	https://bioconductor.org/packages/release/bioc/html/GSVA.html
pROC package (version 1.18.0)	Robin et al.^31^	https://cran.r-project.org/web/packages/pROC/index.html
gProfiler2 (version 0.2.1)	Kolberg et al.^59^	https://biit.cs.ut.ee/gprofiler/gost
UCSC Genome Browser Kent et al.^60^		http://genome.ucsc.edu
Trimmomatic (version 0.36)	Bolger et al.^61^	https://github.com/usadellab/Trimmomatic
Hisat2 (version 2.1.0)	Kim et al.^62^	http://daehwankimlab.github.io/hisat2/
featureCounts of subread (version 1.5.3)	Liao et al.^63^	https://subread.sourceforge.net
DeSeq2 (version 1.38.3)	Love et al.^64^	https://bioconductor.org/packages/release/bioc/html/DESeq2.html
MeRIPTools (version 0.2.1)	Zhang et al.^65^	https://github.com/scottzijiezhang/MeRIPtools/blob/master/DESCRIPTION
RADAR (version 0.2.4)	Zhang et al.^66^	https://rdrr.io/github/scottzijiezhang/RADAR/
deepTools (version 2.0)	Ramirez et al.^67^	deeptools.readthedocs.io
edgeR (version 3.40.2)	Robinson et al.^68^	https://bioconductor.org/packages/release/bioc/html/edgeR.html

## Highlights

- Expression of RNA methyltransferases METTL3/14 correlates with survival in neuroblastoma
- Inhibition of METTL3/14 decreases cell survival and reduces tumor growth in xenograft models
- METTL3/14 inhibition regulates RNA stability by mediating site-specific m^6^A loss
- METTL3/14 inhibition up-regulates differentiation-associated transcriptional networks
