# Supplementary material for: Small-molecule inhibition of the METTL3/METTL14 complex suppresses neuroblastoma tumor growth and promotes differentiation
Source: Cell Rep. Author manuscript; Available in PMC 2024 Jun 17. (PMC11181463; doi:10.1016/j.celrep.2024.114165)
Supplement: 1 [file NIHMS2000776-supplement-1.pdf]

Cell Reports, Volume *43*

Supplemental information

Small-molecule inhibition of the METTL3/METTL14 complex suppresses neuroblastoma tumor growth and promotes differentiation

Monica Pomaville, Mohansrinivas Chennakesavalu, Pingluan Wang, Zhiwei Jiang, Hui-Lung Sun, Peizhe Ren, Ryan Borchert, Varsha Gupta, Chang Ye, Ruiqi Ge, Zhongyu Zhu, Mallory Brodnik, Yuhao Zhong, Kelley Moore, Helen Salwen, Rani E. George, Malgorzata Krajewska, Alexandre Chlenski, Mark A. Applebaum, Chuan He, and Susan L. Cohn

Supplemental Figures

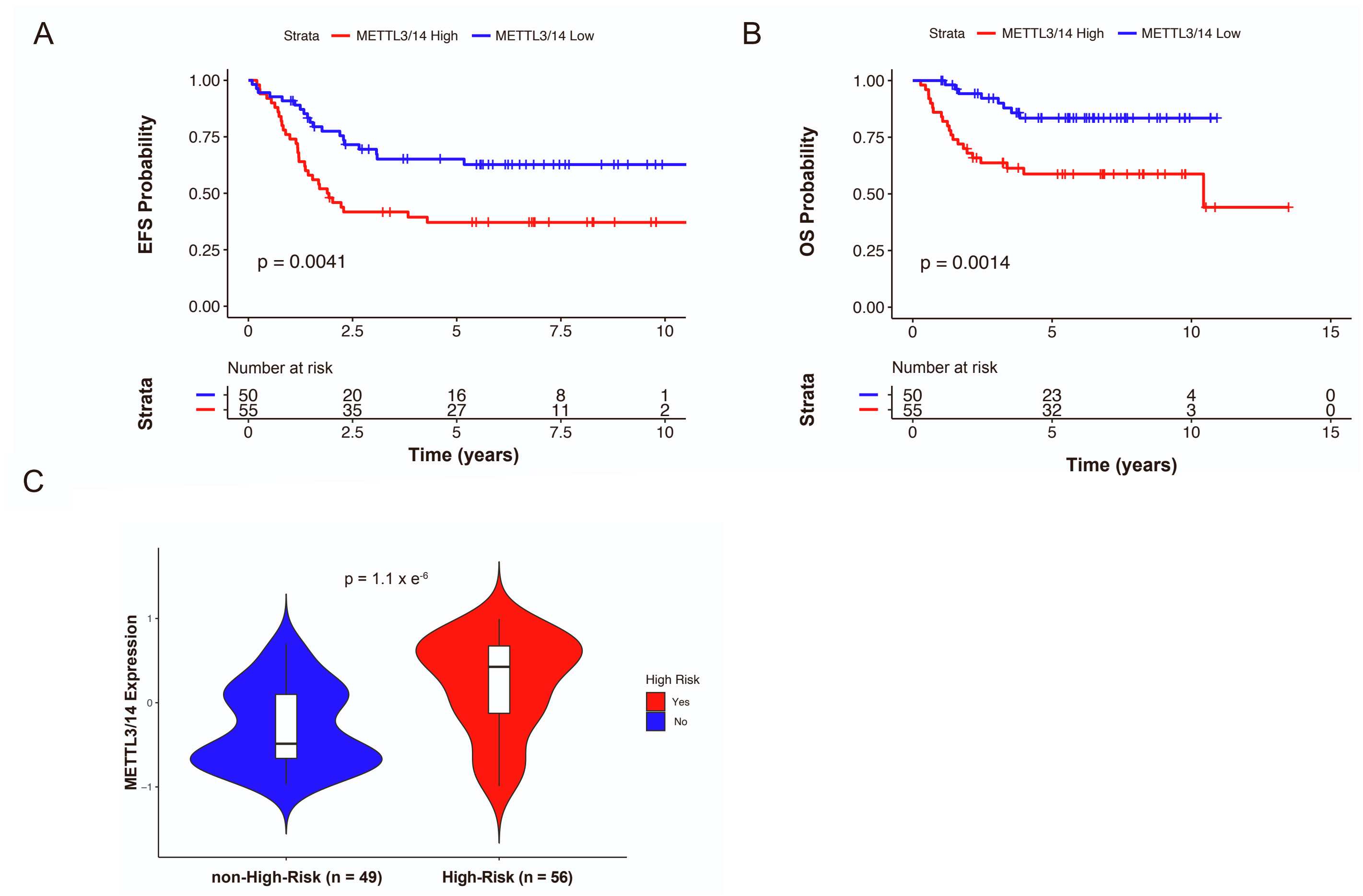

Figure S1. METTL3/METTL14 expression correlates with survival in patients with neuroblastoma. (A) Kaplan Meier survival curve showing event-free survival of patients with neuroblastoma in the Westermann cohort (n=105) according to METTL3/METTL14 expression. **(B)** Kaplan Meier survival curve showing overall survival of patients with neuroblastoma in the Westermann cohort (n=105) according to METTL3/METTL14 expression. **(C)** Violin plot showing relative distribution of METTL3/METTL14 expression in patients in the Westermann cohort classified as having high-risk (n=56) or non-high-risk (n=49) neuroblastoma.

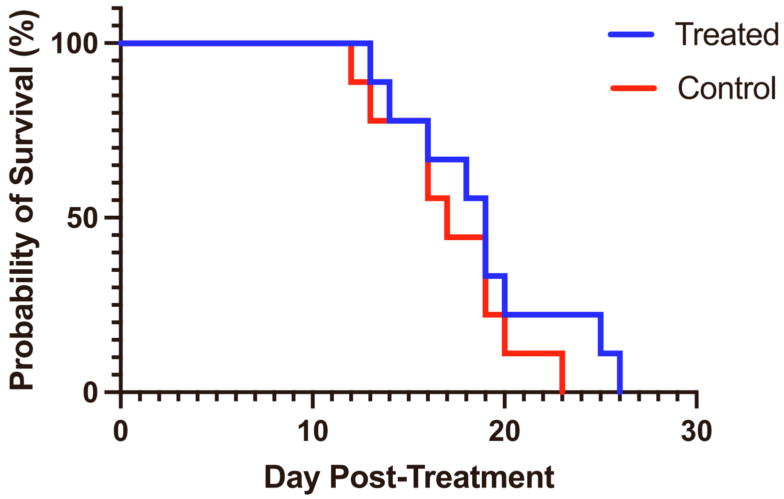

Figure S2. Effect of METTL3/14 inhibition in neuroblastoma xenografts derived from NGP cells. Overall survival of mice with neuroblastoma xenografts derived from NGP cells treated with STM2457 versus vehicle control is not significantly different (p=0.3138).

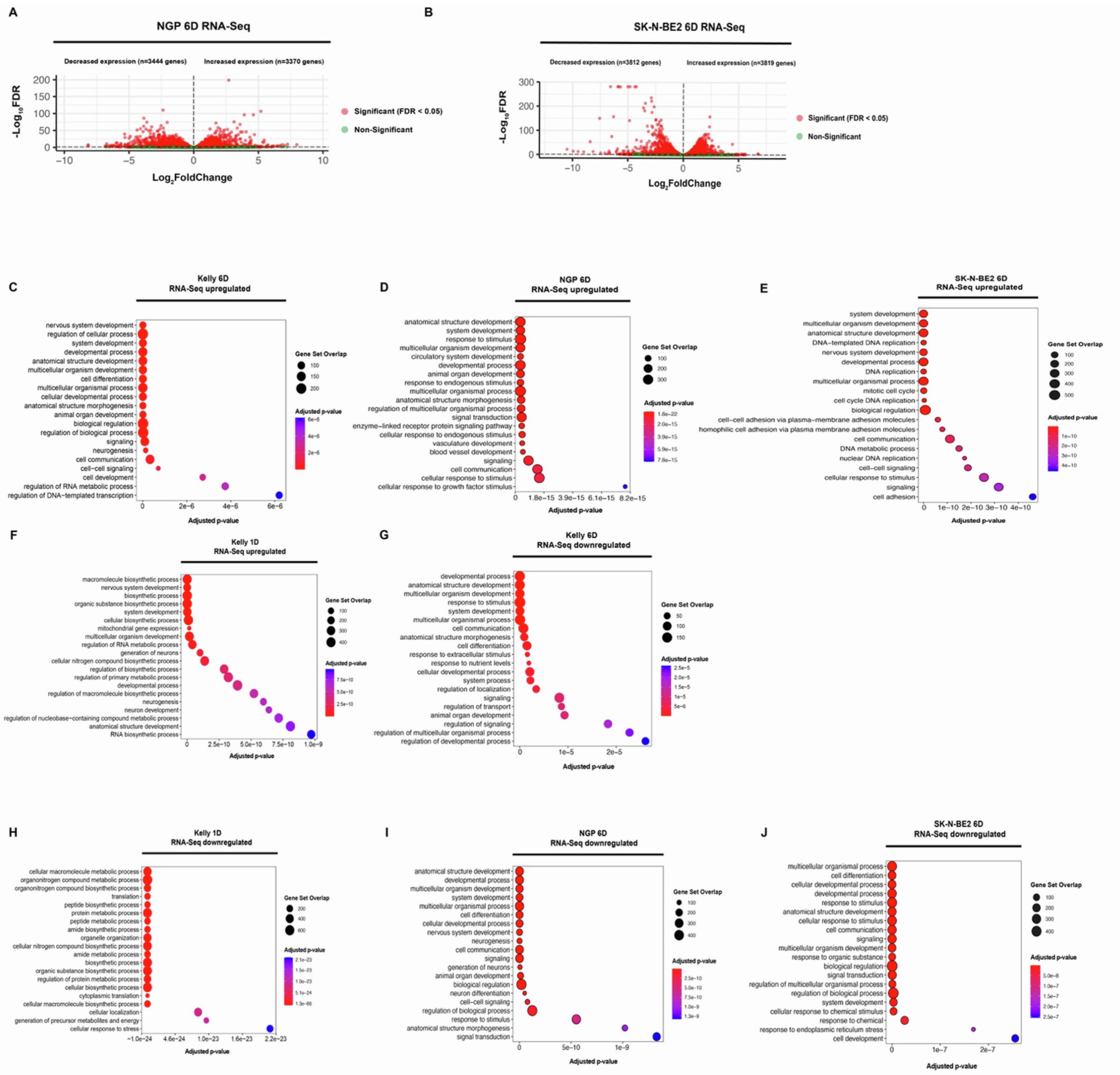

Figure S3. STM2457 treatment increases expression of neuronal differentiation genes. **(A)** Volcano plot showing differentially expressed genes in NGP cells treated for 6 days with 1 µM STM2457. **(B)** Volcano plot showing differentially expressed genes in SK-N-BE2 cells treated for 6 days with 12 µM STM2457. **(C)** GO pathway analysis showing up-regulated gene pathways in Kelly cells, **(D)** NGP cells, and **(E)** SK-N-BE2 cells after 6 days of treatment with STM2457 (8 µM, 1 µM, and 12 µM, respectively). **(F)** GO pathway analysis showing up-regulated gene pathways in Kelly cells treated with 8 µM STM2457 for 1 day. **(G)** GO pathway analysis showing down-regulated gene pathways in Kelly cells treated with 8 µM STM2457 for 6 days, **(H)** Kelly cells treated with 8 µM for 1 day, **(I)** NGP cells treated with 1 µM STM2457 for 6 days, and **(J)** SK-N-BE2 cells treated with 12 µM STM2457 for 6 days.

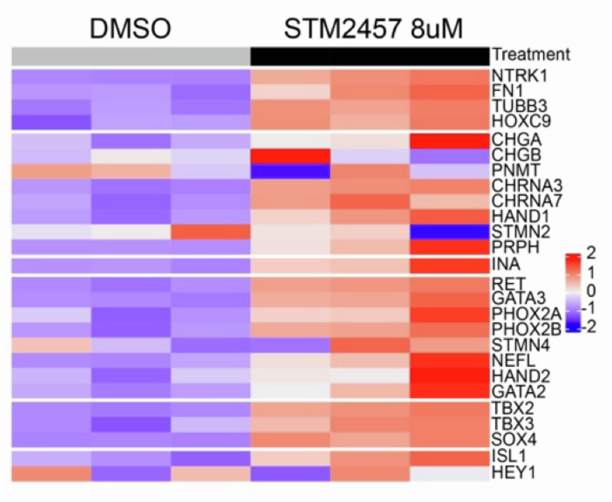

Figure S4. STM2457 treatment promotes expression of selected differentiation genes. Heat map of expression (z scored expression) of a previously defined set of neuronal differentiation genes (Supplementary Table 1) in Kelly cells treated with DMSO or 8 μM STM2457 for six days.

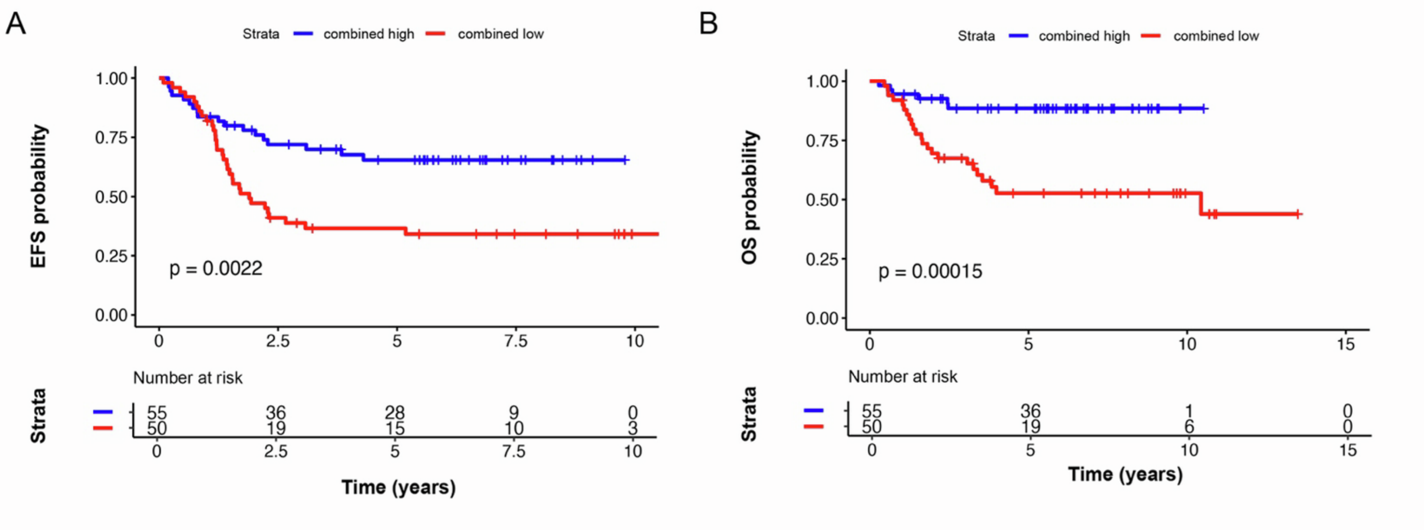

Figure S5. High METTL3/14 inhibitor response signature scores are correlated with improved survival in neuroblastoma. (A) Event-free survival and **(B)** overall survival is significantly improved for patients in the Westermann cohort with high expression of STM2457 targets (n=55) compared to those with low expression (n=50) (p=0.0022 and p=0.00015, respectively). STM2457 targets were defined from the 73 genes in the nervous system development pathway (GO:0007399) that are found up-regulated in NGP, Kelly, and SK-N-BE2 cells after treatment with STM2457 for 6 days (1 μM, 8 μM, and 12 μM, respectively).

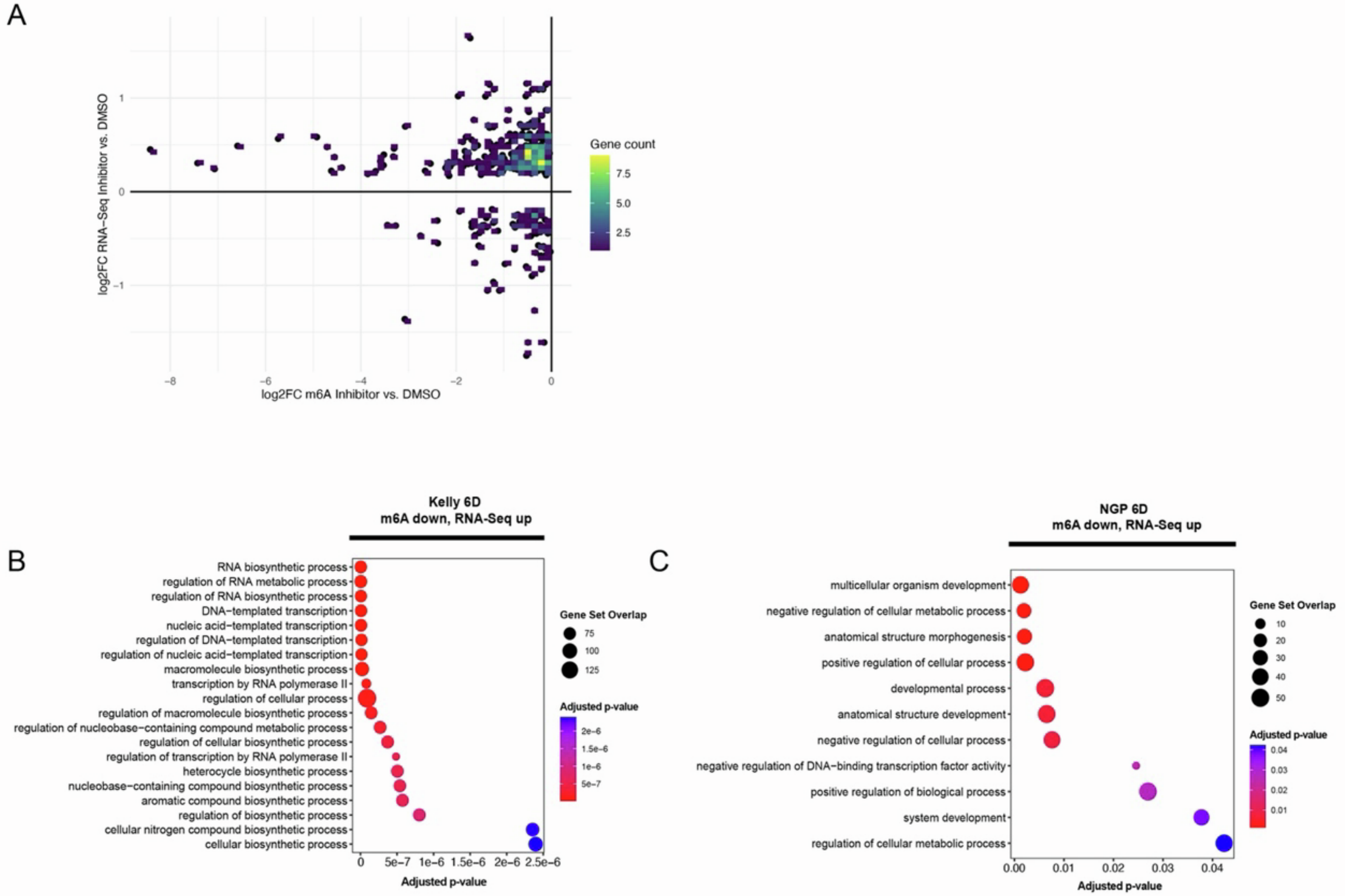

Figure S6. STM2457 treatment decreases m^{6}A mRNA deposition and increases mRNA expression in networks enriched for neuronal differentiation. **(A)** STM2457 treatment of Kelly cells for 6 days promotes loss of mRNA m^{6}A deposition (x axis) assessed by meRIP sequencing and increased gene expression assessed by RNA sequencing (y axis) compared to DMSO. **(B)** GO analysis of upregulated METTL3 targets following treatment with 8μM STM2457 for 6 days in Kelly cells and in **(C)** NGP cells.

A

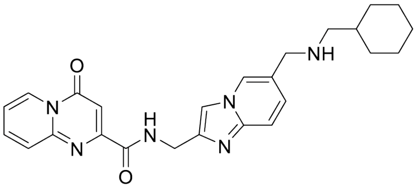

STM2457

B 1H NMR (400 MHz, DMSO) δ 9.16 (t, J = 6.0 Hz, 1H), 9.02 (dd, J = 7.1, 2.1 Hz, 1H), 8.40 (s, 1H), 8.08 (ddd, J = 8.6, 5.3, 1.7 Hz, 1H), 7.80 (d, J = 8.2 Hz, 1H), 7.50 – 7.42 (m, 2H), 7.23 (dd, J = 9.2, 1.8 Hz, 1H), 6.90 (s, 1H), 4.61 (d, J = 6.0 Hz, 2H), 3.70 (s, 2H), 2.36 (d, J = 6.8 Hz, 2H), 1.73 (dd, J = 12.9, 2.6 Hz, 2H), 1.68 – 1.56 (m, 3H), 1.41 (t, J = 7.0 Hz, 1H), 1.22 – 1.05 (m, 3H), 0.89 – 0.83 (m, 2H). 13C NMR (101 MHz, DMSO) δ 162.89, 157.92, 153.67, 150.49, 144.14, 143.46, 138.54, 128.50, 127.43, 126.70, 126.20, 117.33, 116.43, 116.05, 110.85, 100.81, 52.14, 47.95, 37.53, 34.45, 29.93, 25.52, 25.01. HRMS C25H29N6O2+ [M+H]+ calculated 445.2347, found 445.2353.

C

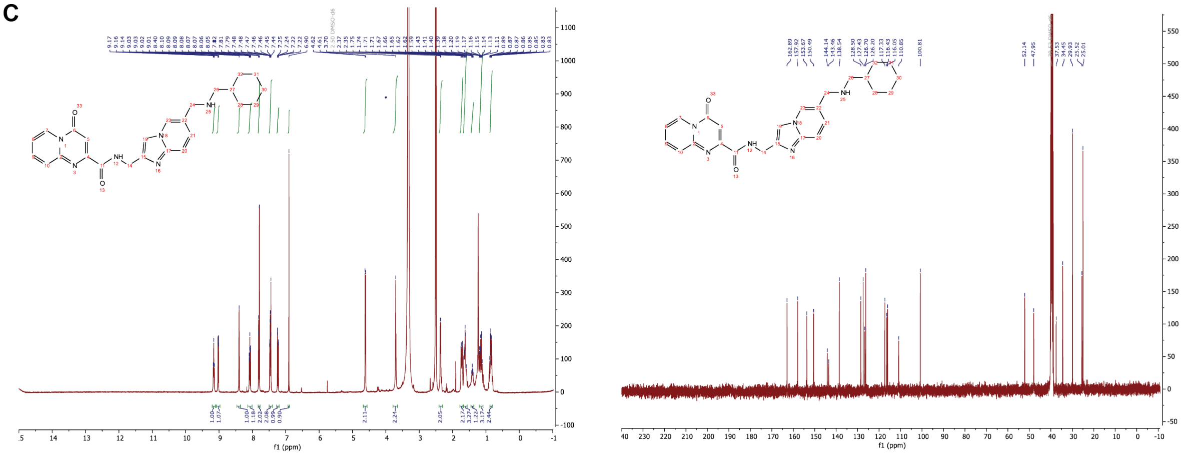

Figure S7. The METTL3/14 inhibitor STM2457 was synthesized and validated. (A) Structure of METTL3/METTL14 inhibitor, STM2457. **(B)** Molecular identification based on Nuclear Magnetic Resonance (NMR) analysis. **(C)** NMR analysis.

Gene Name	Classification 1	Citation 1	Classification 2	Citation 2
**STMN2**	Chromaffin	Bedoya-Reina [1]		
**CHGA**	Chromaffin	Bedoya-Reina [1]	Adrenergic	Bedoya-Reina [1]
**CHGB**	Chromaffin	Bedoya-Reina [1]	Adrenergic	Bedoya-Reina [1]
**HAND1**	Chromaffin	Bedoya-Reina [1]		
**PNMT**	Chromaffin	Bedoya-Reina [1]	Adrenergic	Bedoya-Reina [1]
**CHRNA3**	Chromaffin	Bedoya-Reina [1]		
**NTRK1**	Differentiation	Nakagawara [2]	Adrenergic	Bedoya-Reina [1]
**TUBB3**	Differentiation	Zimmerman [3]		
**HOXC9**	Differentiation	Mao [4]		
**FN1**	Differentiation	Zimmerman [3]		
**INA**	Central Nervous System	Kildisiute [6]	Fetal Medullary	Kildisiute [6]
**RET**	Sympathoadrenal development	Bedoya-Reina [1]		
**STMN4**	Sympathoadrenal development	Bedoya-Reina [1]		
**NEFL**	Sympathoadrenal development	Bedoya-Reina [1]		
**HAND2**	Sympathoadrenal development	Tomolonis [7]		
**GATA3**	Sympathoadrenal development	Tomolonis [7]		
**PHOX2A**	Adrenergic	Bedoya-Reina [1]	Sympathoadrenal development	Tomolonis [7]
**PHOX2B**	Adrenergic	Bedoya-Reina [1]	Sympathoadrenal development	Tomolonis [7]
**GATA2**	Sympathoadrenal development	Tomolonis [7]		
**SOX4**	Retino-sympathetic	Zimmerman [3]		
**TBX2**	Retino-sympathetic	Zimmerman [3]		
**TBX3**	Retino-sympathetic	Zimmerman [3]	Fetal Medullary	Kildisiute [6]
**PRPH**	Sympathoblast	Bedoya-Reina [1]	Chromaffin	Bedoya-Reina [1]
**HEY1**	Adrenergic-type neuroblastoma	Van Groningen [5]		
**CHRNA7**	Progenitor	Bedoya-Reina [1]	Chromaffin	Bedoya-Reina [1]
**ISL1**	Adrenergic	Bedoya-Reina [1]		

Table S1. List of differentiation-associated genes compiled from RNA sequencing in Kelly cells and literature search.

Shared upregulated genes between Kelly, NGP, and SK-N-BE2 cells within the nervous system development pathway (GO:0007399)			
GFRA2	APBA2	LLGL1	KIF14
PLPPR4	CDKN2C	NR0B1	
PLXNA4	ATP7A	CTNNB1	
TMEM108	SFRP1	KDM2B	
DOK4	TBX3	EDNRA	
CNP	MAP4K4	EHMT2	
GPRIN1	OGDH	MEIS1	
MARCKSL1	LRRN3	B4GALT5	
NES	DCHS1	PTPRS	
CHRM1	TUBB2B	MAP1A	
TENM4	GLI2	LPAR1	
PLPPR5	NREP	RTN1	
XRCC2	SDC2	ATXN1	
SULF2	DAG1	PBX1	
MMP2	DLC1	TEAD2	
GRIP1	SYNE2	SMARCC2	
MDGA1	FN1	CHRNA7	
EVL	SPTBN1	CCDC14	
ACTB	TIAM1	PKD2	
GDF11	BASP1	GPR173	
MACF1	ENC1	MTR	
SPG7	COL4A1	DCX	
ST8SIA2	PHACTR4	DPYSL2	
IGF1R	IGSF9	ZEB2	

Table S2. List of genes up-regulated in Kelly, NGP, and SK-N-BE2 cells after treatment with STM2457 (8 μM, 1 μM, 12 μM, respectively) and list of genes up-regulated in Kelly, NGP, and SK-N-BE2 cells after treatment with STM2457 (8 μM, 1 μM, 12 μM, respectively) classified as part of the nervous system development pathway (GO:0007399).

Genes upregulated in STM2457-treated tumors relative to vehicle control after 14 consecutive days of treatment and followed to 23 days of life.				
HSPA1A	SEC61A1	DPP7	RPL23	RPS14
NR4A1	RPL13	LIMD2	BSG	FLNA
MT-CYB	RTL1	SRRM3	GPS1	NUCB1
HSPB1	INTS11	TRMT1	TELO2	PNKP
RPL36	FBRSL1	DPP9	DLGAP4	RPS8
MDK	EEF1A2	UBA52	FAM207A	STRA6
GAPDH	SP100	RPL10	ZNF316	ZDHHC8
RPS11	NOP56	MT-CO3	WDR74	CC2D1A
MT-ND5	CEP131	FAM193B	CHTF18	RPL35
RPS19	CRH	RRP12	DOK4	NPDC1
SEZ6L2	MT-ND6	GADD45GIP1	IGFBP2	RPL32
RPL28	ELOB	MT-CO2	DNAJB1	TTC9B
MT-ND4L	SERF2	KLHL17	PPP6R1	
CHGA	SNRNP70	IGSF9	GATA2	
ACIN1	RPL19	PSMC3	IER5L	
JUN	COL6A1	CDC37	RPS24	
HSPA1B	MAP1B	RPL37	ARFGAP1	
TBX2	RABL6	IER2	PDZD4	
MT-ND3	RPL8	HEXD	SF3A2	
COL6A2	NOP58	MFSD12	RPL4	
RPS27	H1-10	RPLP1	KRT17	
PTMS	PELP1	AHCY	PPDPF	
MT-ND4	RPL18	TNFRSF25	SCRIB	
CCNL2	SAMD1	RPL29	TPM2	
LRRC4B	PRRT2	ANKRD13B	GDPD5	

Table S3. Genes up-regulated in STM2457-treated tumors (50mg/kg/dose) derived from Kelly cells relative to vehicle control after 14 consecutive days of treatment and followed to 23 days of life.

Shared genes that are upregulated, contain lower m^{6}A, and have increased half-life in Kelly cells treated with METTL3/14 inhibitor	
ABHD8	KIF26A
ADRA2A	KIF3C
ALDH1B1	MARVELD1
APC2	MEX3D
ARID1A	MPST
B3GALT6	NEU4
BRPF3	P3H4
C11orf95	PATZ1
CHST2	PCDH1
CKAP4	PLEKHO1
DDN	PPP1R18
DDX17	RBM15B
DOK4	RHOB
FAM171A2	SBK1
FBXO41	SLC10A4
FEM1A	SLIT3
FEN1	SOX12
FLAD1	SOX4
FSCN1	TBX2
GPRIN1	TNRC18
HCFC1	TRIB2
HNRNPF	UBTF
HOXC9	ZNF48
JARID2	ZNF581
KIF18B	ZNF787

Table S4. List of genes assessed by RNA sequencing in Kelly cells after treatment with 8 μM STM2457 that show increased expression, decreased m^{6}A, and increased stability after 6 days of treatment.

Primer sequences used				
Actin	F	CACCATTGGCAATGAGCGGTTC		
Actin	R	AGGTCTTTGCGGATGTCCACGT		
CHGA	F	GGTTCTTGAGAACCAGAGCAGC		
CHGA	R	GCTTCACCACTTTTCTCTGCCTC		
NTRK1	F	CACTAACAGCACATCTGGAGACC		
NTRK1	R	TGAGCACAAGGAGCAGCGTAGA		

Table S5. Primer sequences used in this study.

Supplemental reference list [1-7]

1. Bedoya-Reina, O.C., Li, W., Arceo, M., Plescher, M., Bullova, P., Pui, H., Kaucka, M., Kharchenko, P., Martinsson, T., Holmberg, J., et al. (2021). Single-nuclei transcriptomes from human adrenal gland reveal distinct cellular identities of low and high-risk neuroblastoma tumors. Nat Commun *12*, 5309. 10.1038/s41467-021-24870-7.
2. Nakagawara, A., Arima-Nakagawara, M., Scavarda, N.J., Azar, C.G., Cantor, A.B., and Brodeur, G.M. (1993). Association between high levels of expression of the TRK gene and favorable outcome in human neuroblastoma. N Engl J Med *328*, 847-854. 10.1056/NEJM199303253281205.
3. Zimmerman, M.W., Durbin, A.D., He, S., Oppel, F., Shi, H., Tao, T., Li, Z., Berezovskaya, A., Liu, Y., Zhang, J., et al. (2021). Retinoic acid rewires the adrenergic core regulatory circuitry of childhood neuroblastoma. Sci Adv *7*, eabe0834. 10.1126/sciadv.abe0834.
4. Mao, L., Ding, J., Zha, Y., Yang, L., McCarthy, B.A., King, W., Cui, H., and Ding, H.F. (2011). HOXC9 links cell-cycle exit and neuronal differentiation and is a prognostic marker in neuroblastoma. Cancer Res *71*, 4314-4324. 10.1158/0008-5472.CAN-11-0051.
5. van Groningen, T., Koster, J., Valentijn, L.J., Zwijnenburg, D.A., Akogul, N., Hasselt, N.E., Broekmans, M., Haneveld, F., Nowakowska, N.E., Bras, J., et al. (2017). Neuroblastoma is composed of two super-enhancer-associated differentiation states. Nat Genet *49*, 1261-1266. 10.1038/ng.3899.
6. Kildisiute, G., Kholosy, W.M., Young, M.D., Roberts, K., Elmentaite, R., van Hooff, S.R., Pacyna, C.N., Khabirova, E., Piapi, A., Thevanesan, C., et al. (2021). Tumor to normal single-cell mRNA comparisons reveal a pan-neuroblastoma cancer cell. Sci Adv *7*. 10.1126/sciadv.abd3311.
7. Tomolonis, J.A., Agarwal, S., and Shohet, J.M. (2018). Neuroblastoma pathogenesis: deregulation of embryonic neural crest development. Cell Tissue Res *372*, 245-262. 10.1007/s00441-017-2747-0.
